# Genetic quality assurance and genetic monitoring of laboratory mice and rats: FELASA Working Group Report

**DOI:** 10.1177/0023677219867719

**Published:** 2019-08-20

**Authors:** Fernando Benavides, Thomas Rülicke, Jan-Bas Prins, James Bussell, Ferdinando Scavizzi, Paolo Cinelli, Yann Herault, Dirk Wedekind

**Affiliations:** 1 Department of Epigenetics and Molecular Carcinogenesis, The University of Texas, MD Anderson Cancer Center, USA; 2 Institute of Laboratory Animal Science, University of Veterinary Medicine, Vienna, Austria; 3 The Francis Crick Institute, London, UK; 4 Leiden University Medical Centre, Leiden, The Netherlands; 5 Biomedical and Veterinary Services Department, University of Oxford, Oxford, UK; 6 National Research Council (IBCN), Rome, Italy; 7 Department of Trauma Surgery, University of Zurich, Zurich, Switzerland; 8 Université de Strasbourg, CNRS, INSERM, Institut de Génétique Biologie Moléculaire et Cellulaire, IGBMC, Illkirch, France; 9 Université de Strasbourg, CNRS, INSERM, Institut Clinique de la Souris, CELPHEDIA-PHENOMIN-ICS, Illkirch, France; 10 Institute of Laboratory Animal Science, Hannover Medical School, Hannover, Germany

**Keywords:** Animal facilities, genetics, quality assurance/control, refinement, rodents

## Abstract

Genetic quality assurance (QA), including genetic monitoring (GeMo) of inbred strains and background characterization (BC) of genetically altered (GA) animal models, should be an essential component of any QA programme in laboratory animal facilities. Genetic quality control is as important for ensuring the validity of the animal model as health and microbiology monitoring are. It should be required that studies using laboratory rodents, mainly mice and rats, utilize genetically defined animals. This paper, presented by the FELASA Working Group on Genetic Quality Assurance and Genetic Monitoring of Laboratory Murines, describes the objectives of and available methods for genetic QA programmes in rodent facilities. The main goals of any genetic QA programme are: (a) to verify the authenticity and uniformity of inbred stains and substrains, thus ensuring a genetically reliable colony maintenance; (b) to detect possible genetic contamination; and (c) to precisely describe the genetic composition of GA lines. While this publication focuses mainly on mouse and rat genetic QA, the principles will apply to other rodent species some of which are briefly mentioned within the context of inbred and outbred stocks.

## Résumé

L'assurance qualité (AQ) génétique, dont la surveillance génétique (SG) des souches consanguines et la caractérisation des souches (CS) de modèles animaux génétiquement modifiés (GM) devrait constituer un aspect essentiel de tout programme d'AQ dans les installations de recherche animale. Le contrôle qualité génétique est aussi important que la surveillance sanitaire ou microbiologique pour assurer la validité du modèle animal. Il devrait être obligatoire que les études utilisant des rongeurs de laboratoire, principalement des souris et des rats, utilisent des animaux génétiquement définis. Le document présenté par le groupe de travail FELASA sur l'assurance qualité et la surveillance génétiques des rats et souris de laboratoire, décrit les objectifs et les méthodes disponibles pour les programmes d'AQ menés dans les installations utilisant des rongeurs. Les objectifs principaux d'un programme d'AQ sont les suivants : (i) vérifier l'authenticité et l'uniformité des souches consanguines et des sous-souches, assurant ainsi la maintenance d'une colonie génétiquement fiable; (ii) détecter les contaminations génétiques éventuelles; et (iii) décrire précisément la composition génétique des lignées GM. Bien que cette publication se concentre sur l'AQ génétique des souris et des rats, les principes s'appliqueront à d'autres espèces de rongeurs, dont certaines sont brièvement mentionnées dans le contexte des animaux issus de lignées consanguines ou croisées.

## Abstract (de)

Die genetische Qualitätssicherung (QA), einschließlich des genetischen Monitorings (GeMo) von Inzuchtstämmen und der Hintergrundcharakterisierung (BC) von genetisch veränderten (GA) Tiermodellen, sollte generell ein wesentlicher Bestandteil aller QA-Programme in Versuchstiereinrichtungen sein. Die genetische Qualitätskontrolle ist zur Gewährleistung der Validität von Tiermodellen ebenso wichtig wie die Überwachung ihrer Gesundheit und mikrobiologischen Qualität. Für Studien mit Labornagern, hauptsächlich betrifft es Mäuse und Ratten, sollte ausschließlich die Verwendung von genetisch definierten Tieren vorgesehen werden. Dieses Dokument, das von der FELASA Arbeitsgruppe über genetische Qualitätssicherung und genetisches Monitoring von Labormäusen und -ratten präsentiert wird, beschreibt die Ziele und verfügbaren Methoden für genetische QA-Programme in Labortierhaltungen. Die Hauptziele eines jeden genetischen QA Programms sind: (i) Überprüfung der Authentizität und Uniformität von Inzuchtstämmen und deren Substämme, um so eine genetisch zuverlässige Erhaltung der Kolonie zu gewährleisten, (ii) Erkennung möglicher genetischer Kontaminationen, und (iii) präzise Beschreibung der genetischen Beschaffenheit von GA-Linien. Diese Veröffentlichung konzentriert sich hauptsächlich auf die genetische QA von Maus und Ratte, wobei die Prinzipien auch für andere Nagetierarten, von denen einige im Zusammenhang mit Inzucht- und Auszuchtstämmen kurz erwähnt werden, zutreffen.

## Resumen

La garantía de calidad genética (QA), incluidos el monitoreo genético (GeMo) de las cepas consanguíneas y la caracterización de fondo genético (BC) de los animales genéticamente modificados (GA), debería ser un componente esencial de cualquier programa de *QA* en los animalarios de roedores. El control de la calidad genética es tan importante para asegurar la validez del modelo animal como lo es el control de calidad sanitaria y microbiológica. Debería exigirse que los estudios que utilicen roedores de laboratorio, principalmente ratones y ratas, utilicen exclusivamente animales genéticamente definidos. Este manuscrito, presentado por *FELASA Working Group on Genetic Quality Assurance and Genetic Monitoring of Laboratory Murines*, describe los objetivos y métodos disponibles para los programas de calidad genética en instalaciones de roedores de laboratorio. Los principales objetivos de cualquier programa de calidad genética son: (i) verificar la autenticidad y uniformidad de las cepas (y sub-cepas) consanguíneas; (ii) detectar una posible contaminación genética; y (iii) describir con precisión la composición genética de las líneas genéticamente modificadas. Si bien esta publicación se centra principalmente en los controles de calidad genética de ratones y ratas, los mismos principios se aplican a otras especies de roedores de laboratorio, algunas de las cuales se mencionan brevemente en el contexto de las cepas consanguíneas y los grupos exocriados de ratones y ratas.

## Standardized laboratory rodents

### Inbred strains

The International Committee on Standardized Genetic Nomenclature for Mice and The Rat Genome Nomenclature Committee considers a strain inbred

if it has been propagated by systematically mating brothers to sisters (or younger parent to offspring) for 20 or more consecutive generations, and individuals of the strain can be traced to a single ancestral pair at the twentieth or subsequent generation.

At this point, animals within the population will average ≤2% residual heterozygosity, and the individuals may be regarded as genetically identical (isogenic).^1^ However, it has been estimated that 24 generations of sib-mating are needed to reach a heterozygosity rate < 1% and 36 generations to reach (almost) complete isogeneity.^2^

Isogeneity implies *histocompatibility*, meaning the strains are syngeneic. Syngeneic animals will permanently accept tissue transplants from any individual of the same strain and sex. Unlike cloned animals and monozygotic twins (which are 100% identical for all genomic loci), inbred rodents, besides being isogenic, are also homozygous at almost all genomic loci. Overall, each inbred strain represents a unique, although fortuitous, assortment of alleles.^3^ If a strain were to be remade from scratch, using the same founders, after the same 20 generations of inbreeding it would create a genetically distinct strain due to the random assortment and fixation of alleles. Baseline phenotypic data for the most common inbred mouse strains are available through a coordinated international effort initiated by The Jackson Laboratory and implemented through The Mouse Phenome Database (http://phenome.jax.org/).^4^ An example of baseline phenotypic data is presented in Supplementary Tables 1A and 1B. The Mouse Genome Informatics (MGI) website^5^ provides a list, compiled by Dr Michael Festing (http://www.informatics.jax.org/external/festing/search_form.cgi), of 420 inbred mouse and 230 inbred rat strains (some of which have been lost or terminated), along with brief descriptions. The list includes widely used inbred mouse strains: A/J, BALB/c, C3H/He, C57BL/6, DBA/2, FVB/N and others; and rat strains: ACI, BN, F344, LE and WKY.

### Outbred stocks

Outbred stocks are populations of laboratory animals that differ from inbred strains in that they are genetically heterogeneous. Compared with inbred strains or F1 hybrids, the genetic constitution of a given animal, taken randomly from an outbred stock, is not known a priori. However, all of the animals in the group share group characteristics (identity), such as being albino (although not all outbred mice or rats are albino), good breeders and relatively tame compared to other strains; features that make these animals very popular as foster mothers for assisted reproductive techniques. Examples of outbred stocks of mice are ICR (CD-1), CFW and NMRI (all derived from the original ‘Swiss’ mice imported to the USA by Clara J. Lynch in 1926) and (non-Swiss) CF-1. Examples of outbred rat stocks are Sprague Dawley (SD), Wistar (WI) and Long-Evans (LE). Since outbred stocks are not genetically defined, quality control is commonly based on assessing expected phenotypic traits, such as coat colour, growth and reproductive characteristics, based on data from the large colonies of commercial breeders. Because outbred colonies, like human populations, are heterogeneous, they are frequently used in toxicology and pharmacology research.^6^ However, several geneticists have disputed this use and have criticized studies in which outbred mice were used inappropriately, wasting both animal lives and precious resources in suboptimal experiments.^7^

### Other standardized strains of mice and rats

**Figure 1.**
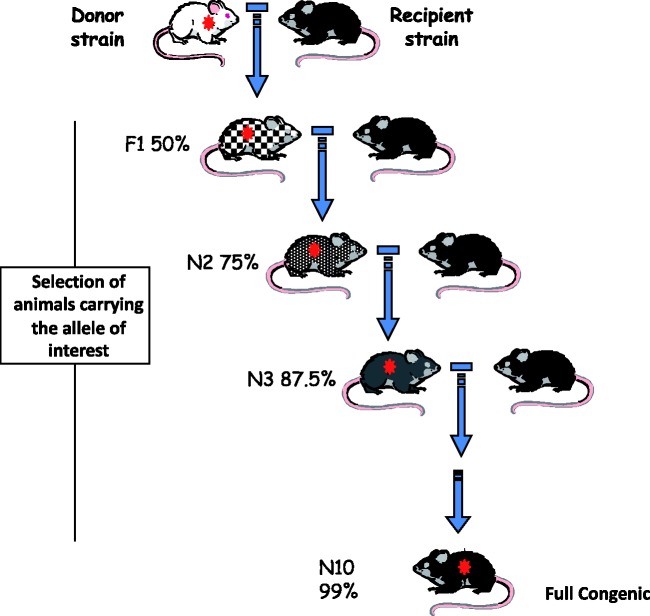
This scheme represents the successive steps in the establishment of a congenic strain. The initial step is a cross between the donor strain (albino in the example) carrying the gene of interest (e.g. a targeted gene or a transgene) and a recipient or background strain (black in the example). At each generation, a breeder carrying the gene of interest (*) is backcrossed to a partner of the recipient strain (genetically linked genes are transferred with it and the size of the introgressed fragment can be many thousands or millions of bases, and include many genes). The degree of grey colour indicates that, after each backcross generation, the offspring have an increased amount of the background genome (average percentage is indicated in each N generation). When the modified gene is not resulting in an easily recognizable phenotype (e.g. skin or behavioural changes), molecular genotyping is necessary to select the carrier (heterozygous) mice.

*F1 hybrids* result from the outcross of two separate inbred strains and are heterozygous at all loci for which the parental strains harbour different alleles. F1 littermates are genetically identical and are histocompatible. *Congenic strains* are produced by crossing two strains: the *donor strain* that carries the allele or chromosomal region of interest, and the *recipient* or *background strain* that will receive the locus of interest. F1 offspring generated by crossing donors and recipients are then backcrossed to the recipient strain. Offspring that carry the allele of interest are identified and again backcrossed to the background strain. This process is typically repeated for 10 or more successive generations (Figure 1), unless marker-assisted backcrosses (speed congenics) are used. Repeated backcrossing results in the chromosomes of the background strain progressively replacing those of the donor strain, except for a chromosomal region that carries the allele of interest.

## Genetically altered (GA) rodents

Before presenting the different types of GA rodents, it is worth mentioning that there are basically two different approaches to characterizing gene function. *Forward genetics* (from phenotype to genotype) aims to characterize the gene alteration that is responsible for a specific mutant phenotype (typically from spontaneous or chemically-induced mutations). *Reverse genetics* is the opposite approach and aims to characterize the function of a gene by analysing the consequences (at the phenotypic level) of alterations normally engineered by researchers at the DNA level. This section introduces the four basic types of GA rodents, those created by: (a) pronuclear microinjection, (b) vector- mediated transgenesis (c) homologous recombination in embryonic stem (ES) cells, (d) gene editing nucleases, and (e) either chemically induced or spontaneous mutations. Detailed descriptions of the technologies used to create GAs have been published.^8^ Before selecting a gene-editing technique to create a genetically modified animal, it is important to check an appropriate database such as those hosted by The Jackson Laboratories and the International Mouse Phenotyping Consortium as to whether a suitable animal model already exists (see Supplementary Table 2 for the complete list of online resources for laboratory mouse and rat strains).

### Transgenesis by pronuclear microinjection

Transgenic mice were introduced in the early 1980s^9^ and were the first *transgenic animals*. It is advisable to use the term ‘transgenic’ only for animals whose genomes have been altered by the random insertion of DNA. (There are numerous terms used to describe genetic changes in animals: genetically engineered mice (GEM) or genetically modified mice (GMM) are typically used to describe any type of genetic modification in the mouse. We use the term GA rodent here to also include those carrying spontaneous or chemically induced mutations, and ‘line’ instead of ‘strain’ for GA rodents.) Transgenic rodents are almost exclusively created by the pronuclear microinjection of foreign DNA fragments directly into one of the two pronuclei of one-cell embryos (zygote), a technique that is still widely used. In this process of *additive transgenesis*, the microinjected transgene randomly integrates into the genome as a single copy or more often as a concatemer with variable copy number. The mouse and rat models created with this system typically express or, in the resultant concatemer, overexpress a transgene placed under the control of a tissue-specific, developmental-stage-specific, or ubiquitous promoter (along with other regulatory elements), all contained in the transgene DNA construct.

The recommended generic symbol for a transgenic insertion is Tg. The founder transgenic animals are hemizygous for the DNA segment and are designated Tg/0. Transgenes are extra segments of DNA that have no corresponding ‘wild-type’ sequence in the unmodified homologous chromosome in hemizygous animals, that is why the use of ‘0’ instead of ‘+’ (typically used to denote wild-type alleles) is recommended. Each transgenic line generated via random integration creates a unique animal model and each putative founder must be developed independently. Traditionally, to distinguish between homozygous (Tg/Tg) and hemizygous (Tg/0) mice, the mouse of interest was crossed to a non-transgenic partner and the progeny were statistically analysed for Mendelian segregation of the transgene. A more modern technique uses quantitative real-time polymerase chain reaction (qPCR) to distinguish hemizygous from homozygous transgenic mice.^10^ In order to achieve a pure genetic background (recommended), the transgene must be introduced into embryos derived from an inbred strain.

A later improvement on the constructs used in the transgenesis approach was the introduction of inducible systems in which transgene expression can be turned on and off. Examples of this strategy are the Tet-on and Tet-off expression systems. In these systems, transcription of a given transgene is placed under the control of a tetracycline-controlled trans-activator protein, which can be regulated, both reversibly and quantitatively, by exposing the transgenic mice to either Tetracycline (Tc) or one of its derivatives, such as Doxycycline (Dox). Both Tet-on and Tet-off are binary systems that require the generation of double transgenic (*bigenic*) mice.^11^

### Vector-mediated transgenesis

Alternative methods for transgenesis by random integration are based on vectors of different origin. Most important and very efficient are retroviral/lentiviral vectors^12^ and transposons.^13^ Also pre-treated spermatozoa have been successfully used as vectors in combination with ICSI (intracytoplasmic sperm injection).^14^ Each technique has advantages and disadvantages and the corresponding principle of transgene integration may affect the quality of the resulting GA models. Viral vectors and transposons for instance integrate as a single copy, however multiple integrations, randomly distributed in the genome, are not uncommon. Major concerns exist regarding the impact of sperm-mediated gene transfer on the sperm genetic material, possibly induced by the pre-treatment of spermatozoa.^15^

### Targeted mutagenesis by homologous recombination using ES cells

Another important technology utilizes murine ES cell lines. ES cells are undifferentiated, pluripotent, embryonic cells derived from the inner cell mass of pre-implantation blastocysts that can participate in forming the germ-cell lineage of chimeric mice, an indispensable step in generating founder mice carrying the targeted mutation. Historically, the first ES cell lines were derived from embryos of the 129 family (129S2, 129P3, etc.), that is inbred strains originally bred for the isolation of embryonic carcinoma (EC) cells. Today ES cell lines are available from many mouse strains and those of the C57BL/6N origin have become widespread and are often selected for trans-national projects (e.g. EUCOMM).

In cases where constitutive null alleles lead to complex phenotypes, reduced viability, or have other drawbacks, conditional alleles may be used, allowing one to control the time and tissue where a gene is turned off, typically using the Cre/*lox*P system.^16^ Production of conditional KOs requires two independent lines: one providing a source of Cre recombinase, an enzyme derived from bacteriophage P1, in the tissue under study, and another containing *lox*P (locus *o*f *X*-ing over *P*1) sites flanking the DNA segment of interest that needs to be crossed to generate double mutant mice. The Cre enzyme cuts and recombines the ‘*floxed*’ DNA at *lox*P sites. The Cre transgene can be made inducible, adding more sophistication to the system. The tamoxifen-inducible Cre^ERT2^ which can be activated in a spatio-temporal manner by administration of tamoxifen, is widely used.^17^ The Cre-*lox*P strategy can also be used to regulate the expression of reporter genes. For example, the *lac*Z gene can be driven by a ubiquitous promoter (e.g. *Rosa 26*) with a floxed stop sequence, containing several terminator codons inserted between the promoter and the *lac*Z coding sequence.

### Gene editing using nucleases

Over the last 10 years, a number of new techniques have been developed for the production of targeted mutations using engineered nucleases. These techniques, briefly described here, provide ES cell-independent methods to create targeted mutations in laboratory mice, rats and other species.

To make mutations using zinc-finger nucleases (ZFN), two complementary and sequence-specific multi-finger peptides containing the *FokI* nuclease domain must be designed. Each peptide is designed to recognize a specific DNA sequence spanning 9–18 base pairs (bp) on either side of a 5–6 bp sequence, which defines the targeted region. When injected into a pronucleus or cytoplasm of zygotes, the ZFN assemblies bind tightly, one on each strand, on both sides of the target site. The dimerized *FokI* endonuclease then creates double strand DNA breaks (DSBs) triggering cellular mechanisms to repair the damage. Damage is normally repaired by either homology-directed repair (HDR) or non- homologous end joining (NHEJ). HDR requires a homologous template to guide the repair and thus re-establishes the original sequence. NHEJ is much less precise and cause nucleotide deletions that lead to frame shifts that create potential loss-of-function or truncation mutations. Mice and rats carrying null alleles or sequence-specific modifications have already been produced using ZFN technology.^18^ Like ZFNs, transcription activator-like effector nuclease (TALEN) technology involves the combination of a nonspecific DNA endonuclease fused to a DNA-binding domain, but can be more easily engineered (compared to ZFN) to target a particular DNA sequence.

The CRISPR (clusters of regularly interspaced short palindromic repeats)/Cas system, commonly implemented as CRISPR/Cas9, is based on a primitive defence mechanism that allows bacteria and archaea to fight against infection from viruses, plasmids and phages.^19^ CRISPR-based guide RNAs (gRNAs) are designed to target a Cas endonuclease to cut DNA at the desired site through RNA-guided DNA cleavage. The RNA-guided endonucleases can be engineered to cleave virtually any DNA sequence by appropriately designing the gRNA, for example to generate KO mice.^20^ CRISPR/Cas technology has several advantages over ZFNs and TALENs. The main advantage is the ease of design and the flexibility of using a sequence-specific RNA interacting with the Cas enzyme instead of a complex sequence-specific protein (DNA-binding domain) fused to a nuclease. Also, mutations in multiple genes can be generated in a single step by injecting mice with multiple gRNAs that simultaneously target different genes.^21^ Such multiplex gene editing has been successful in cells, as well as mouse and rat embryos.^20^ CRISPR/Cas9 has been used to create insertions, deletions and point mutations. The system is highly flexible, fast and efficient, and is revolutionizing genomic engineering in mammals.^22^ It allows making KO and KI lines in any genetic background. DNA can be electroporated (with size restrictions) or injected into either the cytoplasm or pronuclei of 1-cell or 2-cell stage embryos, thus avoiding the use of ES cells and chimeras. However, as each engineered animal is unique, this technology requires extensive sequence analysis to characterize multiple putative founders to ensure the presence of the desired mutation and the absence of undesired on- and off-target mutations or unpredictable larger genome alterations,^23,24^ while also identifying mosaic founders (G0). Once identified, the selected founder should be bred with wild-type animals to evaluate transmission of the mutation.

### Spontaneous and chemically-induced mutations

A list of GA rodent types is not complete without including both spontaneous and chemically-induced mutations. Spontaneous mutations, generally identified through the observation of an abnormal phenotype, present several advantages. First and foremost, they are produced at virtually no cost and are generally freely available. Second, they usually have an obvious phenotype, as they are identified based on observation. Third, spontaneous mutations represent a great variety of molecular events, such as deletions, insertions and point mutations, generating not only loss-of-function alleles but also hypomorphic and hypermorphic alleles. Finally, mutations arise in a variety of backgrounds including inbred strains and outbred stocks. Several spontaneous mutations have provided rodent models for human conditions. These include classical mutations such as, *nude* (*Foxn1*^*nu*^), *scid* (*Prkdc*^*scid*^), *hairless* (*Hr*^*hr*^), *diabetes* (*Lepr*^*db*^), *obese* (*Lep*^*ob*^) and *X-linked muscular dystrophy* (*Dmd*^*mdx*^) in the mouse; and the mutations behind the Rowett nude (*Foxn1*^*rnu*^) and Zucker diabetic fatty (*Lepr*^*fa*^) models in the rat.

The discovery of the extraordinary virtues of the alkylating agent N-ethyl-N-nitroso urea (ENU) as a mutagen was a milestone in the history of mouse genetics. Researchers using ENU have generated and propagated numerous mutant alleles for protein-coding genes, thus establishing a precious tool for genome annotation. Because ENU typically creates point mutations, it has been widely used in forward genetic screens. The major drawback of ENU-induced mutagenesis is that it creates random mutations rather than targeted mutations. Several projects have been undertaken to systematically and extensively phenotype the offspring of ENU-mutagenized males. Large ENU mutagenesis programmes have been conducted in Germany, England and the USA.^25^

### Quality assurance and exchange of GA-rodents

#### What to ensure after (in-house) generation or upon arrival?

The possibility of crossing different GA lines combined with the increasing complexity of targeting approaches has greatly increased the number of available GA models. The need to cross different GA lines together for a particular study generates additional complexity, especially at the genetic background level. Many mutants have been and are still generated on a hybrid genetic background. Therefore, it is essential to keep adequate records of detailed information for all genetically modified strains. This information must be transferred with the strain to all collaborators and users. The most important information includes the correct strain name, a complete description of the mutation, the genetic background of the animals, a genotyping protocol and observed phenotypic changes. Together, these provide the minimum information for the recommended ‘rodent-passport’, and several forms have been designed for cataloguing this information. We recommend the data sheet developed by the FELASA Working group on the refinement of methods for genotyping genetically modified rodents.^26^

Every mutant strain name must provide precise information on the affected gene, the type of mutation and the genetic background. For in-house generated strains, one must provide a specific Institute for Laboratory Animal Research Laboratory (ILAR) Code Registration for the laboratory where the mutant originated. An overview on the importance of nomenclature can be found in the ‘FELASA guidelines for the production and nomenclature of transgenic rodents’.^27^ A name designed according to the international nomenclature rules is the only means to unambiguously distinguish strains from each other. This is important when the same strain is held in different facilities around the world and/or they are listed in archives and databases. Further, it is imperative that strains be properly described in publications using a universal nomenclature. Without a common nomenclature, it becomes impossible to accurately communicate scientific results. Vague or incomplete names create errors rendering experiments irreproducible.

## Origin and consequences of genetic variation

A serious challenge facing rodent animal facilities is keeping inbred strains genetically pure and GA lines on a defined background. Changes in the genetic constitution of inbred strains can be produced by (a) contamination by accidental outcrosses and (b) genetic drift due to residual heterozygosity or fixation of *de novo* spontaneous mutations.

### Genetic contamination

The accidental mating of individuals from one inbred strain with animals of another origin is by far the most important source of genetic profile alteration in inbred strains. Genetic contamination of this type, which always results in a sudden and massive exchange of alleles, is more likely between strains that have similar coat colour (i.e. albino (*Tyr*^*c*^/*Tyr*^*c*^), agouti (*A/A*), or non-agouti (*a/a*)). Where lines have the same coat colour alleles, extra care must be taken when housing them in close proximity of each other.

### Spontaneous mutations and polymorphisms

Spontaneous mutations are a source of uncontrolled genetic variation that is often impossible to detect by simple phenotypic observation or routine genetic monitoring (GeMo). *Genetic polymorphism* is the presence of alternative DNA sequences (alleles) at a locus among individuals, groups, or populations, at a frequency >1%. Two types of genetic markers are commonly used in association studies and genetic quality control: microsatellites and single nucleotide polymorphisms (SNPs) (see ‘Marker systems’ below).

### Genetic drift and the generation of substrains

While permanent inbreeding effectively eliminates a proportion of new mutant alleles, another undetected fraction may become progressively fixed in the homozygous state, replacing the original allele, a process known as *genetic drift*. Genetic drift contributes inexorably to strain divergence and the generation of substrains when the same strain is propagated independently in different places.^28^ Examples of mouse substrains are abundant, for example there are *c*. 10 documented BALB/c substrains and *c*. 15 C57BL/6 substrains including the J and N substrains from The Jackson Laboratory (Jax) and the National Institutes of Health (NIH), respectively.^29^ In the same way, many rat inbred strains present at least two substrains, for example SHR has at least four substrains (including SHR/Ola and SHR/NCrl), and WKY and F344 have at least three substrains each. Substrain variability has been confirmed by sequencing analysis for these rat substrains,^30^ with WKY showing the highest degree of substrain variation (this is in part due to the supply of the model prior to the prescribed 20 generation inbreeding requirement).

### Undesirable passenger mutations

Mutations that are hidden in the genomes of substrains or GA lines and can affect the outcome of an experiment are sometimes referred to as *passenger mutations*.^31^ There are many examples in the literature where substrains originating from the same inbred strain have acquired new phenotypes as a consequence of genetic drift.^32^ For example, mice of the C57BL/6JOlaHsd substrain are homozygous for a deletion of the α-synuclein (*Snca*) and multimerin (*Mnrn1*) genes.^33,34^ Likewise, some spontaneous mutations differentially segregate in C57BL/6J and C57BL/6N, the most common substrains of C57BL/6, separated in 1951. These include a retinal degeneration mutation in the *Crb1* gene (*Crb1*^*rd8*^), present only in the N substrain, and a deletion in the *Nnt* gene, present only in the J substrain.^35,36^ Berghe and colleagues recently reported that passenger mutations are also common in most GA lines derived from 129 ES cells, and that these mutations persist even after the creation of fully congenic strains.^37^ This is not trivial; Berghe et al. estimated that close to 1000 protein-coding genes could be aberrantly expressed in the 129-derived chromosomal segments that are still segregating in these congenic lines. This finding emphasizes the need for properly chosen control animals to identify phenotypes due to background mutations or the combination of background mutations and the genetic modification of interest, rather than the modification itself.

## Importance of using standard nomenclature

Rules guiding nomenclature were established by the International Committee on Standardized Genetic Nomenclature for Mice and Rats and are continuously updated. These rules, last revised in January 2016, are described on the MGI webpage under ‘Guidelines for Nomenclature of Mouse and Rat Strains’ (http://www.informatics.jax.org/mgihome/nomen/strains.shtml). A helpful and visual Mouse Nomenclature Quick Guide is available at https://www.jax.org/jax-mice-and-services/customer-support/technical-support/genetics-and-nomenclature#. For more details on nomenclature refer to the Supplementary material.

## Genetic quality control programmes

The current gold standard for genetic quality control of laboratory rodents depends on polymorphic *genetic markers* to distinguish between different genetic backgrounds. Genetic markers are specific DNA sequences with a known location on a chromosome and are essential tools for genetic quality control. Genetic quality control is essential to determine the genetic composition of an animal and to screen for uniformity and authenticity of a strain. Please note that outbred colonies cannot be tested for authenticity. Instead, the colony is screened for its level of genetic heterogeneity to detect genetic contamination and to monitor the progress of breeding programmes and to select future breeders.

### Marker systems

Many polymorphisms have been described in the mouse and rat; however, only microsatellites and SNPs are used as genetic markers in current QA programmes. Microsatellite markers, also known as Simple Sequence Length Polymorphisms (SSLPs) or Short Tandem Repeats (STRs), are still used in modern GeMo programmes because they are inexpensive and easy to type.^38,39^ Animals are genotyped by analysing PCR-products amplified from short, tandemly arranged, repeating DNA sequences. These repeats are typically 2–6 bp long and are repeated a few to dozens of times creating allelic diversity among stains. Genomic DNA primers are designed to unique sequences flanking the repeats. The PCR products, typically around 100–300 bp in size, are analysed using agarose or polyacrylamide gel electrophoresis. The MGI webpage has comprehensive SSLP information, including primer sequences and size variations in bp for several inbred mouse strains (http://www.informatics.jax.org/marker). A collection of mapped, highly polymorphic, SSLP markers for inbred laboratory rat strains is available in The National BioResource Project – Rat database and is linked to the Map Report of the Rat Genome Database (RGD) (http://rgd.mcw.edu). See Supplementary Table 2 for the complete list of online resources for laboratory mouse and rat strains.

SNP genotyping is an alternative to microsatellites that is now widely used for GeMo. SNP genotyping is inexpensive and can be performed in most research institutions or outsourced. SNPs are the most common genetic variation and exist in both coding and non-coding regions. Almost all SNPs are bi-allelic, presenting one of only two possible nucleotides (e.g. homozygous G/G or T/T), or both (e.g. heterozygous G/T) in an individual. Petkov and co-workers from The Jackson Laboratory have described the allelic distribution of 235 SNPs in 48 mouse strains and selected a panel of 28 SNPs sufficient to characterize the majority of the *c*. 300 inbred, wild-derived, congenic, consomic and recombinant inbred strains maintained at The Jackson Laboratory.^40^ Several publications have reported useful SNPs for the rat. For example, Zimdahl and colleagues described a map with >12,000 gene-based SNPs from transcribed regions.^41^

### GeMo of inbred strains and outbred stocks

Most GeMo techniques used currently are based on microsatellites or SNPs. However, GeMo should not rely solely on molecular techniques, but should take a broader view that includes phenotypic parameters such as coat colour, behaviour and breeding performance. Commercial breeders are extremely sensitized to the risk of genetic contamination and regularly monitor their strains for genetic contamination, but not necessarily genetic drift, by using different sets of SNPs to monitor their nucleus colonies. The Jackson Laboratory incorporated a unique, patented, Genetic Stability Program^42^ designed to effectively limit cumulative genetic drift by rebuilding their foundation stocks from pedigreed, cryopreserved embryos every five generations. For example, starting in 2005, they began selling only C57BL/6J mice derived from two chosen mice through hundreds of frozen embryos of the duo's grandchildren (enough to last for 25–30 years). It should be noted that when recovering strains from frozen stocks good GeMo should be carried out to assure oneself that genetic contamination or wrong genotypes were not present prior to freezing.

For outbred stocks, GeMo helps preserve the genetic heterogeneity and allele pool of a colony. This complex process requires analysing a large number of animals and comparing this data with historical data documenting the alleles present, their frequency and the level of heterozygosity in that particular colony. In some cases, the results can reveal a loss of genetic variability resulting in a colony with very low heterogeneity. The degree of genetic heterogeneity in outbred colonies depends on their history. Low heterogeneity can result from poor selection of future breeding stock, deviation from approved (rotational) breeding systems or the bottleneck effect caused by a small breeding pool, as is common when a small group of breeders is imported or being used to rederive a colony. In contrast, high heterogeneity can result from a recent outcross. In general, outbred stocks are characterized by measuring phenotypic traits and calculating the corresponding mean and standard deviations. Essentially, genetic control of outbred stocks is directed at avoiding inbreeding and stabilizing genetic diversity over many generations.

#### GeMo of inbred mice and rats bred in-house

The best recommendation here is to purchase animals from reliable vendors and replace the breeding stock with animals from the same vendor after 10 generations, rather than to maintain independent colonies of classical inbred strains. As an additional benefit, using animals from the same vendor prevents the formation of substrains harbouring potential mutations and maintains a similar microbiome. Nevertheless, in-house colonies should always be tested with a small set of informative microsatellite markers or SNPs to confirm integrity.

##### Using a small panel of microsatellites (SSLPs)

Microsatellites can be used to verify that the animals in an inbred colony are essentially pure, with no traces of genetic contamination. This is especially important in facilities that maintain strains with the same coat colour in the same room, a particularly dangerous practice especially when not using individually ventilated cage (IVC) systems. Microsatellite testing can normally be performed in-house. The number of markers to use for testing has not been standardized: each situation and facility differs in how many and which strains are kept. Nonetheless, a panel of 40 polymorphic SSLPs, evenly distributed across the autosomes, will rule out recent genetic contamination, if the markers can distinguish among the strains being analysed. Supplementary Table 3 presents a small set of mouse SSLPs that could be used to authenticate some classical inbred strains.

**Figure 2.**
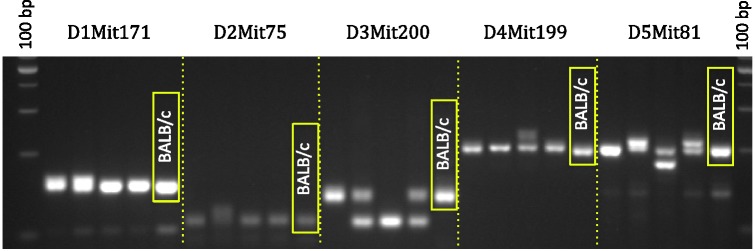
Example of genetic contamination detected by SSLP PCR. The picture shows a 4% agarose gel with the characteristic bands obtained after PCR amplification using genomic DNA from four mice supposedly belonging to the BALB/c strain (first four lanes), plus a standard DNA control for BALB/c (last lane). In this example, only five SSLP loci are shown, located in chromosomes 1 to 5. Note the presence of heterozygosity (two bands) and homozygosity for bands that do not match the standard for BALB/c. This is a clear case of loss of authenticity due to genetic contamination. The PCR products are compared with a 100 bp DNA ladder.

Interpreting SSLP data is straightforward. Because inbred animals are isogenic and homozygous, they will present only one band in the electrophoresis gel, representing a single allele, when genotyped for a particular SSLP. The presence of any heterozygosity, indicated by two bands, or bands that do not coincide with those of the strain control DNA, should be considered as indicating potential strain contamination (Figure 2). How frequently colony strain identity should be evaluated depends on the size of the colony, the generation interval, etc. Generally, testing once every two years is reasonable for a facility maintaining a small number of colonies well-separated in terms of coat colour, and with low numbers of importations.

**Figure 3.**
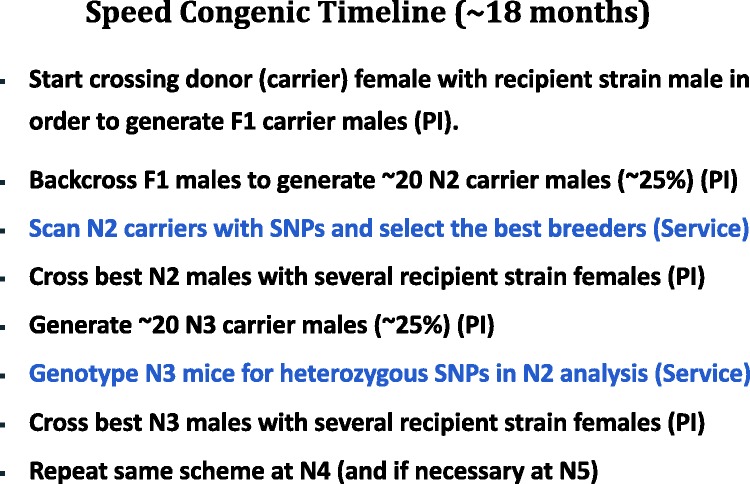
This chart explains the typical timeline for a marker assisted (speed congenic) backcross process. The prediction of >98% recipient genome at N5 is based on the use of 20 best breeders (carriers) at each generation,^55^ however, this number is not always available and fewer breeders can be used, with disparate results, depending also on chance. PI: Principal Investigator (laboratory). Service: the laboratory providing the genome scan with SNP markers.

##### Using a small panel of SNPs

For GeMo purposes only, 40 polymorphic SNPs, evenly distributed across the chromosomes is a reasonable number for detecting recent genetic contamination (this suggestion should be modified dependent on the conditions or risks in each facility). SNP genotyping is currently available on different platforms, that vary in cost and automation capabilities. Kompetitive Allele Specific PCR (KASP), a variation on allele-specific PCR, uses allele-specific oligo extension and fluorescence resonance energy transfer,^43^ has the advantage that it can be automated using 96- or 384-well plates and pipetting robots for the PCR reactions (Supplementary Figure 3). Another option, real-time PCR (TaqMan®) technology, uses specific primers coupled with a sequence-specific, fluorescent TaqMan probe, is effective and easy to automate; however, the cost per individual assay is expensive compared with KASP assays, and requires a more costly real-time thermocycler. Finally, microarray-based SNP genotyping is not typically used for small scale, in-house GeMo, but may be an option for vendors of inbred mice. When using or requesting microarray genotyping services, be aware that only a percentage of the SNPs will be polymorphic between the strains under analysis (e.g., *c*. 40% for some classical inbred strain combinations). Information regarding which alleles (C, G, A or T) to expect for a particular SNP/strain combination, and their genomic location are available for hundreds of thousands of SNPs and for the common mouse and rat inbred strains in easily accessed databases and genome browsers (Supplementary Table 2).

#### GeMo of outbred colonies

GeMo of outbred stocks is much more complex, because these animals are not genetically uniform. Outbred colonies are essentially a group of closely related animals, with shared ancestors and group identity, but that exhibit some level of genetic heterozygosity. Since outbred colonies form a population rather than a strain, it is difficult to establish a standard GeMo programme with only a few genetic markers. However, with an adequate number of SNPs or SSLPs, allele frequencies within the population could indicate the identity of the stock.^44^ One of the main problems of in-house outbred stocks is that they are often maintained with a very small number of animals in the breeding colony, causing a reduction of alleles represented in the population. This may impact genetic drift and increase the inbreeding coefficient. Such colonies are neither truly outbred nor inbred. Although SSLPs or SNPs can be used to estimate the level of heterozygosity within the colony, if it is not possible to keep an appropriate number of breeders, it is better to purchase outbred rodents from vendors that maintain a very large colony and use recommended breeding schemes to reduce inbreeding.

### Background characterization (BC) for GA and mutant lines

The explosion in the number of GA lines is exacerbating the problem of undefined ‘mixed backgrounds’ in experimental rodents. This is particularly worrisome for inducible and conditional models that require the crossing of two independent lines (e.g. Cre-expressing lines crossed with floxed lines). Given that genetic background influences phenotype, especially through the influence of modifier genes; mutations, both spontaneous and induced, transgenes, and targeted alleles that are introgressed into a new background may not exhibit the expected phenotype.^45,46^ One of the first cases reporting this phenomenon involved the classical diabetes (*Lepr*^*db*^) mutation that presented transient diabetes in a C57BL/6 background but overt diabetes in C57BLKS.^47^ Other examples include background effects on survival rate in *Egfr* (epidermal growth factor receptor) KO mice^48^ and tumour incidence in *Pten* KO mice.^49^ For this reason, every GA line should be characterized in terms of their genetic background. Moreover, the knowledge of the genetic background of a mutation is also important for the selection of the corresponding control animals.^50^

Genetic markers evenly distributed and covering the entire genome can be used in a genome scan to estimate the percentages of genome coming from different inbred origins. This process of BC is provided by some commercial enterprises and institutional core facilities. A typical BC employs polymorphic markers to distinguish between the suspected inbred strains. In most mouse cases, these strains are C57BL/6 and 129 substrains because, historically, the ES cells used for the development of KO and KI mice through homologous recombination (section “Targeted mutagenesis by homologous recombination using ES cells” above) were derived exclusively from 129 substrains^51^ whereas WT C57BL/6 females were typically used to prove germline transmission from the chimeras. Without subsequent backcrosses, this scheme resulted in a B6;129 mixed background. However, the availability of ES cell lines derived from other strains (particularly from C57BL/6) and the arrival of genome editing techniques (section “Gene editing using nucleases”) that allow direct production of targeted alterations in any mouse or rat strain^52^ is slowly changing this scenario. In any case, the problem of mixed background can be circumvented altogether by (a) injecting transgenes or nucleases (Cas9-sgRNA) into inbred embryos from the strain of choice; (b) modifying the gene of interest in ES cells from the preferred background strain (e.g. using C57BL/6 ES cells); and (c) crossing chimeras and KO/KI founders with mice of the same strain as the ES cells used for the targeting. Finally, if the GA line has already been developed or acquired from a collaborator or repository, a BC should be performed, and if needed, a fully congenic strain should be developed, either by classical or marker-assisted backcrossing. Periodic backcrossing of a congenic strain to the background strain (of reputable source) also minimises divergence and keeps the congenic strain genetically close to the strain background of control animals.

### Marker-assisted backcrossing for quality assurance and refinement

The use of DNA markers has allowed for a much more rapid and rigorous process of congenic strain development called *marker-assisted* backcrossing or *speed congenics*.^53^ This process relies on using polymorphic genetic markers covering the whole genome to determine the percentage of donor genome present in the animals, then selecting the animals with the lowest percentage of donor DNA for the next backcross to the recipient strain. This relies on the regions between the polymorphic genetic markers being those of the donor genome: the denser the number of markers the higher the donor genome can be inferred. Common practice is the use of 100–300 markers. This process reduces the number of generations to reach full congenicity (e.g. from N10 to N5), and therefore strain development time, by approximately half. Using marker-assisted backcrosses and the right number of animals we can obtain *c*. 80% recipient background at N2, *c*. 94% at N3, and *c*. 99% at N4 (compared to the classical mean values of 75.0, 87.5 and 93.7%). A flowchart depicting a standard speed congenic protocol is shown in Figure 3. Ideally, the backcross procedure is started with a donor female and a recipient male. Then, F1 mutant males will carry the correct Y-chromosome and after mating to a recipient female, males of the N2 generation will carry the correct X- and Y-chromosome of the recipient strain (avoiding the use of genetic markers on these chromosomes).^54^ It was predicted by Markel et al. that if 20 best breeders (carriers) are used at each generation of the speed congenics protocol >98% recipient genome can be attained at N5.^55,56^ However, the chromosomal segments flanking the selected locus tend to remain associated with it and this is a limitation of the congenic lines due to the potential presence of modifier genes in this segments, the so-called ‘flanking gene problem’.^57^

## Genetic stability and cryopreservation programmes

For inbred, co-isogenic and congenic strains, breeding methods and genetic stability programmes help to minimize substrain divergence due to genetic drift, and also to prevent genetic contamination by accidental crosses with other strains. To reduce genetic drift, the number of generations of in-house breeding should be minimized, and the lines submitted to repositories such as, JAX, EMMA, MMRRC, IMSR or RIKEN, to be archived as frozen embryos and/or sperm. This secures the line and provides a means of replacing the breeding stock every 10 generations as recommended by The Jackson Laboratory Genetic Stability Program (GSP) in order to slow down cumulative genetic drift.^42^ For outbred stocks, the intent is to minimize inbreeding, maintain heterozygosity and manage genetic drift that would otherwise lead to colony divergence. Ideally, outbred colonies should be maintained with ≥25 breeding pairs, all of which have to contribute to the next generation, in order to avoid an increase of the inbreeding coefficient per generation of more than 1%. Smaller colonies drift fast toward homozygosity because breeders are closely related.^58^

Cryopreservation strategies have been adopted for long-term storage of embryos and gametes in several large centralized repositories including the EMMA/INFRAFRONTIER (European Mutant Mouse Archive), the Knock Out Mouse Project (KOMP) Repository, The Jackson Laboratory Repository, The Center for Animal Resources and Development (CARD) and the Riken Bio Resource Center, which can provide cryopreserved material or live mice to laboratories. These repositories facilitate the availability of these strains to the worldwide scientific community and provide a backup for a potential loss of a strain. The International Mouse Strain Resource (IMSR) is a searchable online database of mouse strains, stocks and mutant ES cell lines available worldwide, including inbred, mutant and genetically engineered strains (http://www.findmice.org/).

## Supplemental Material

Supplemental material for Genetic quality assurance and genetic monitoring of laboratory mice and rats: FELASA Working Group Report Click here for additional data file. Supplemental Material for Genetic quality assurance and genetic monitoring of laboratory mice and rats: FELASA Working Group Report by Fernando Benavides, Thomas Rülicke, Jan-Bas Prins, James Bussell, Ferdinando Scavizzi, Paolo Cinelli, Yann Herault and Dirk Wedekind in Laboratory Animals

## Supplementary Material

The full report, including 160 references, is available as Supplemental Material to this publication online via https://journals.sagepub.com/doi/full/10.1177/0023677219867719.
