# Supplementary material for: Genetic quality assurance and genetic monitoring of laboratory mice and rats: FELASA Working Group Report
Source: Lab Anim. 2019 Aug 20;54(2):135–48. doi: 10.1177/0023677219867719 (PMC7160752; doi:10.1177/0023677219867719)
Supplement: Supplemental material for Genetic quality assurance and genetic monitoring of laboratory mice and rats: FELASA Working Group Report [file Supplemental_Material.pdf]

GENETIC QUALITY ASSURANCE AND GENETIC MONITORING OF LABORATORY MICE AND RATS. FELASA WORKING GROUP REPORT

Fernando Benavides (convener)[1], Thomas Rülicke[2], Jan-Bas Prins[3,4], James Bussell[5], Ferdinando Scavizzi[6], Paolo Cinelli[7], Yann Herault[8,9], and Dirk Wedekind[10]

[1]Department of Epigenetics and Molecular Carcinogenesis, The University of Texas MD Anderson Cancer Center, 1808 Park Road 1C, Smithville, Texas 78957, USA.

[2]Institute of Laboratory Animal Science, University of Veterinary Medicine Vienna, Veterinärplatz 1, A-1210 Vienna, Austria.

[3] The Francis Crick Institute, 1 Midland Road, London NW1 1AT, United Kingdom.

[4]Leiden University Medical Centre, P.O. Box 9600, 2300 RC Leiden, The Netherlands.

[5]University of Oxford, Biomedical and Veterinary Services Department, Mansfield Road, Oxford OX1 3TA, United Kingdom.

[6]National Research Council (IBCN), CNR-Campus International Development (EMMA-INFRAFRONTIER-IMPC), via E. Ramarini 32, I-00015 Monterotondo Scalo, Roma, Italy.

[7]University of Zurich, University Hospital Zurich, Department of Trauma Surgery, Sternwartstrasse 14, CH-8091 Zurich, Switzerland.

[8]Université de Strasbourg, CNRS, INSERM, Institut de Génétique Biologie Moléculaire et Cellulaire, IGBMC, 1 rue Laurent Fries, Illkirch, France.

[9]Université de Strasbourg, CNRS, INSERM, Phenomin, Institut Clinique de la Souris (ICS), 1 rue Laurent Fries, Illkirch, France.

[10]Institute of Laboratory Animal Science, Hannover Medical School, 30625, Hannover, Germany.

Corresponding Author:

Fernando J. Benavides, Department of Epigenetics and Molecular Carcinogenesis, The University of Texas MD Anderson Cancer Center, 1808 Park Road 1C, Smithville, TX, 78957, USA. Email: fbenavid@mdanderson.org

Table of Contents

Preface ... *4*

1. Standardized laboratory rodents ... *5*

1.1 Inbred strains ... **5**

1.2 Outbred stocks ... **8**

1.3 F1 Hybrids ... **10**

1.4 Other standardized strains of mice and rats ... **11**

2. Genetically altered (GA) rodents ... *13*

2.1 Transgenesis by pronuclear microinjection ... **14**

2.2 Vector mediated transgenesis ... **17**

2.3 Targeted mutagenesis by homologous recombination using ES cells ... **17**

2.4 Gene editing using nucleases ... **20**

2.4.1 Zinc-finger nucleases and transcription activator-like effector nucleases ... 21

2.4.2 The CRISPR/Cas System ... 22

2.5. Spontaneous and chemically induced mutations ... **23**

2.6. Quality assurance and exchange of GA-rodents ... **24**

2.6.1. What to ensure after (in-house) generation or upon arrival? ... 24

2.6.2. Description of the genetic modification ... 26

2.6.3. Quality assurance for genotyping samples ... 27

2.6.4. The challenges of using CRISPR/Cas9 systems ... 28

3. Origin and consequences of genetic variation ... *29*

3.1 Genetic contamination ... **29**

3. 2 Spontaneous mutations and polymorphisms ... **30**

3.3 Genetic drift and the generation of substrains ... **30**

3.4 Undesirable passenger mutations ... **32**

4. Importance of using standard nomenclature ... *33*

5. Genetic quality control programmes ... *35*

5.1 Marker Systems ... **36**

5.1.1 Microsatellite markers ... 37

5.1.2 Single Nucleotide Polymorphisms (SNPs) ... 38

5.2 Genetic monitoring (GeMo) of inbred strains and outbred stocks ... **39**

5.2.1 GeMo of inbred mice and rats bred in-house. ... 42

5.2.2 Discrimination of substrains ... 47

5.2.3 GeMo of outbred colonies ... 48

5.3 Background characterization (BC) for GA and mutant lines ... **49**

5.4 Marker-assisted backcrossing for quality assurance and refinement ... **52**

6. Genetic stability and cryopreservation programmes *54*
7. Management of rodent facilities *57*
7.1 Facility organization **58**
7.2. Assisting programmes (ART, cryopreservation, and re-derivation) **59**
7.3 Working according to protocol (Standard Operating Procedures) **60**
8. Education *64*
9. Sources and further reading *66*
10. References *68*

Preface

Genetic quality assurance (QA), including genetic monitoring (GeMo) of inbred strains and background characterization (BC) of genetically altered (GA) animal models, should be an essential component of any QA programme in laboratory animal facilities. Genetic quality control is as important for ensuring the validity of the animal model as health and microbiology monitoring are. It should be required that studies using laboratory rodents, mainly mice and rats, utilize genetically defined animals. This manuscript, presented by the FELASA Working Group on Genetic Quality Assurance and Genetic Monitoring of Laboratory Murines, describes the objectives of and available methods for genetic QA programmes in rodent facilities. The main goals of any genetic QA programme are: (i) to verify the authenticity and uniformity of inbred stains and substrains, thus ensuring a genetically reliable colony maintenance; (ii) to detect possible genetic contamination; and (iii) to precisely describe the genetic composition of GA lines. While this publication focuses mainly on mouse and rat genetic QA, the principles will apply to other rodent species some of which are briefly mentioned within the context of inbred and outbred stocks.

1. Standardized laboratory rodents

1.1 Inbred strains

The International Committee on Standardized Genetic Nomenclature for Mice and The Rat Genome Nomenclature Committee considers a strain inbred '*if it has been propagated by systematically mating brothers to sisters (or younger parent to offspring) for 20 or more consecutive generations, and individuals of the strain can be traced to a single ancestral pair at the twentieth or subsequent generation*'. At this point, animals within the population will average ≤ 2% residual heterozygosity, and the individuals may be regarded as genetically identical (isogenic) [1]. However, it has been estimated that 24 generations of sib-mating are needed to reach a heterozygosity rate <1% and 36 generations to reach (almost) complete isogeneity [2]. Most inbred mouse and rat strains commonly used in research have gone through tens of generations of inbreeding. Some strains have been bred in this manner since the beginning of the last century, meaning for over 200 generations (for example, in 2018 DBA/2J reached F224).

Isogeneity implies *histocompatibility,* meaning the strains are syngeneic. Syngeneic animals will permanently accept tissue transplantations from any individual of the same strain and sex. Unlike cloned animals and monozygotic twins (which are 100% identical for all genomic loci), inbred rodents, besides being isogenic, are also homozygous at almost all genomic loci (polymorphic in the founder ancestors). This

is because after a few tens of generations, one allele segregating at a given locus becomes fixed, whereas the others are lost. Fixation occurs when one allele, present at generation F, is absent in at least one of the two breeders mated to produce generation F + 1 causing its permanent loss. Which alleles become fixed or lost usually depends on chance. Overall, each inbred strain represents a unique, although fortuitous, assortment of alleles [3]. If a strain were to be remade from scratch, using the same founders, after the same 20 generations of inbreeding it would create a genetically distinct strain due to the random assortment and fixation of alleles.

The most striking consequence of fixation of alleles in inbred mice is the diverse coat colours of distinct strains. More important, however, are induced physiological changes, which can either benefit specific research applications or confound and negate experimental results. Each inbred strain has a unique collection of characteristics that should be carefully considered when selecting an animal model. For example, homozygosity at particular alleles renders some strains blind (e.g., the $\textit{Pde6b}^{\textit{rd1}}$ mutation) or causes age related hearing loss (e.g., the $\textit{Cdh23}^{\textit{ahl}}$ mutation). Some strains are susceptible to spontaneous or induced tumour development, whereas others are resistant to tumour formation. Some strains are aggressive and others are relatively tame, and so on. Baseline phenotypic data for the most common inbred mouse strains are available through a coordinated international effort initiated by The Jackson Laboratory and implemented through The Mouse Phenome Database [4] (http://phenome.jax.org/). An example of baseline phenotypic data is

presented in **Tables 1A and 1B**.

The Mouse Genome Informatics (MGI) website [5] provides a list, compiled by Dr Michael Festing (http://www.informatics.jax.org/external/festing/search_form.cgi), of 420 inbred mouse and 230 inbred rat strains (some of which have been lost or terminated), along with brief descriptions. The list includes widely used inbred mouse strains A/J, BALB/c, C3H/He, C57BL/6, DBA/2, FVB/N, and others, and rat strains ACI, BN, F344, LE, and WKY. Since the publication of the first drafts of the mouse (C57BL/6J) [6] and rat (BN/SsNHsd) [7] genome sequences, several mouse and rat genomes have been sequenced, including A/J, BALB/cJ, C3H/HeJ, C57BL/6NJ, DBA/2J, FVB/N, and NOD/ShiLtJ mouse genomes, and DA, F344, LE, and SHR rat genomes. Very recently, the Mouse Genomes Project at The Wellcome Trust Sanger Institute in the UK added 36 inbred strain sequences [8]. Notably, classical laboratory mouse strains are somewhat artificial; they do not derive from a single *Mus* genus subspecies [9]. Recent estimates indicate classical inbred strains were predominantly derived from *M. m. domesticus* (~94%), with variable contributions from *M. m. musculus* (~5%) and *M. m. castaneus* (<1%) subspecies [10]. In contrast, all laboratory rat strains have been derived from only *Rattus norvegicus*. Few inbred strains exist for other laboratory rodents. Examples are the Syrian hamster (*Mesocricetus auratus*) LSH/N strain, the guinea pig (*Cavia porcellus*) classical 2/N and 13/N strains, and the gerbil (*Meriones unguiculatus*) MON/Tum strain.

The two main sources for inbred mouse strains are The Jackson Laboratory (USA) and the Riken Bioresource Centre (Japan). The largest collections of laboratory rats are the Rat Resource & Research Center (RRRC, USA) and The National BioResource Project-Rat (NBRP-Rat, Japan). Comprehensive information about the genetics and the biology of the most common strains, describing their specific genotypes and phenotypes, are available online [11] [12] [13] [14] [15] [16] (websites listed in **Table 2**). In addition, MouseMine and RatMine are continuously updated data warehouses that encompass a variety of source databases making integrated data connecting genes to phenotypes more readily accessible.

1.2 Outbred stocks

Outbred stocks are populations of laboratory animals that differ from inbred strains in that they are genetically heterogeneous. According to the standard definition, outbred stocks are '*closed populations (for at least four generations) of genetically variable animals that are bred to maintain maximum heterozygosity*'. Compared with inbred strains or F1 hybrids, the genetic constitution of a given animal, taken randomly from an outbred stock, is not known *a priori*. However, all of the animals in the group share characteristics (identity), for example being albino (although not all the stocks are albino), good breeders, and relatively tame; features that make these animals very popular as foster mothers for assisted reproductive techniques. Examples of outbred stocks of mice are ICR (CD-1), CFW, and NMRI (all derived from the original 'Swiss' mice imported to the USA by Clara J. Lynch in 1926) and (non-

Swiss) CF-1. Examples of outbred rat stocks are Sprague Dawley (SD), Wistar (WI), and Long-Evans (LE). In contrast to inbred strains, which are usually well characterized and described in databases of research centres, there is no comparable source of detailed information (for example, allele frequencies of specific selected markers) for outbred mice or rats. Since outbred stocks are not genetically defined, quality control is commonly based on assessing expected phenotypic traits, such as coat colour, growth, and reproductive characteristics, based on data from the large colonies of commercial breeders.

Outbred animals are typically bred to maintain a defined level of population heterozygosity and to avoid inbreeding [17]. Several breeding schemes for rigorous outbreeding have been developed and should be applied instead of experimenting with random mating. Frequently used systems include the so called “rotational breeding” [18] [19]. A key element of these schemes is dividing the colony into a fixed number of equally sized groups, determined by which females and males will be mated to each other. A constant number of progeny per parent of the breeding stock is selected to avoid unintended directional selection: the greater the number of breeders in a stock, the smaller the variations in allele frequencies (genetic drift) at each generation [18]. Typically, a large number of breeding pairs (≥ 25) is recommended to serve as founders for maintaining outbred stocks. To minimize the chance of random genetic drift and allele loss, an effective population size (N_e) of at least 400 animals is needed [20].

Because outbred colonies, like human populations, are heterogeneous, they are frequently used in toxicology and pharmacology research [21]. However, several geneticists have disputed this use and have criticized studies in which outbred mice were used inappropriately, wasting both animal lives and precious resources in suboptimal experiments [22]. For these and other applications, a 'synthetic' population, created by crossing inbred strains, can replace any outbred stock. Nonetheless, outbred mouse and rat stocks might be useful to refine Quantitative Trait Loci (QTL) mapping, because over time, these heterogeneous stocks accumulate recombination breakpoints creating 'fine-grained mosaics' that are useful for high-resolution mapping of complex traits [23]. One such model is the Diversity Outbred (DO) stock [24], a genetically diverse mouse resource created from the same founder strains as the Collaborative Cross (see Section 1.4 below). Other investigators recently claimed that outbred mice might be better subjects for some biomedical research [25].

Outbred stocks of other laboratory rodents are also available, including guinea pig, Syrian hamster, Chinese hamster (*Cricetulus griseus*), gerbil, cotton rat (*Sigmodon hispidus*), and sand rat (*Psammomys obesus*).

1.3 F1 Hybrids

F1 hybrids result from the outcross of two separate inbred strains and are heterozygous at all loci for which the parental strains harbour different alleles. F1

littermates are genetically identical (isogenic) and are histocompatible. They will permanently accept tissue transplantations from both parental strains, from their littermates, and from their offspring; however, the parental strains will not accept a graft from the F1 hybrids. F1 hybrids exhibit hybrid vigour, making them an attractive choice in some protocols, for example, DNA pronuclear microinjection (e.g., B6D2F1 mice). However, when they are intercrossed, the resulting F2 generation is genetically heterogeneous due to segregation of polymorphic loci.

1.4 Other standardized strains of mice and rats

In the last few decades, there has been a dramatic increase in the use of *congenic strains*, particularly for the maintenance of transgenes and mutant genes, including knockouts (KO), knockins (KI), and spontaneous mutations. Congenic strains are produced by crossing two strains: the *donor strain* that carries the allele or chromosomal region of interest, and the *recipient* or *background strain* that will receive the locus of interest. F1 offspring generated by crossing donors and recipients are then backcrossed to the recipient strain. Offspring that carry the allele of interest are identified and again crossed to the background strain. This process is typically repeated for 10 or more successive generations (**Figure 1**), unless marker-assisted backcrosses (speed congenics) are used. Repeated backcrosses result in the chromosomes of the background strain progressively replacing those of the donor strain, except for a chromosomal region that carries the allele of interest. For this particular chromosome, the chromosomal segment containing the allele of interest is

reduced in size only when a recombination event occurs that replaces a piece of chromosome of the donor strain with the homologous segment of the background strain. Consequently the chromosomal segments flanking the selected locus tend to remain associated with it and this is a limitation of the congenic lines due to the potential presence of modifier genes in this segments, the so-called "flanking gene problem" [26].

Notably, when a mutation occurs in the breeding nucleus of an inbred strain, the new strain differs from the original only by that specific mutation. These two strains are said to be *co-isogenic*. Co-isogenic strains are extremely useful for gene annotation because they allow a comparison of the phenotypes of two allelic forms of a particular gene without influence from the genetic background. The albino C57BL/6-Tyr^c strain is a co-isogenic strain that is popular for making easily recognizable, chimeric mice from C57BL/6 ES cells injected into albino C57BL/6-Tyr^c/Tyr^c blastocysts [27].

There are several other types of strains, used almost exclusively by geneticists. For example, *consomic strains*, also designated chromosome substitution strains (CSS), are a variation of the congenic strains concept. Here, the introgressed DNA is a complete chromosome, rather than a piece of chromosome flanking a given gene [28]. *Recombinant inbred strains* (RIS) are used mainly for gene mapping and are developed by crossing two parental inbred strains to generate F1 hybrids and then

by intercrossing the F1s to generate F2s. Finally, randomly chosen F2 animals are sib mated for twenty or more generations to develop a group of related inbred strains [29]. RIS derived from the same parental strains are grouped into sets. For example, the C57BL/6 × DBA/2 (BXD) is currently the largest mouse RI set and has ~90 strains. The HXB and BXH rat sets (derived from SHR and BN-Lx strains) are also available for genetic studies. The Collaborative Cross is a variation on the RIS concept and is being established by crossing eight founder strains, thus providing a much higher power of resolution and level of genetic diversity than current RIS sets. The Collaborative Cross will provide a new population model designed for analysing complex traits and diseases by covering 90% of the known genetic variation in laboratory mice (The Complex Trait Consortium) [30 31].

2. Genetically altered (GA) rodents

Before presenting the different types of GA rodents, it is worth to mention that there are basically two different approaches for characterizing gene function. *Forward genetics* (from phenotype to genotype) aims to characterize the gene alteration that is responsible for a specific mutant phenotype (typically from spontaneous or chemically-induced mutations). *Reverse genetics* is the opposite approach and aims to characterize the function of a gene by analysing the consequences (at the phenotypic level) of alterations normally engineered by researchers at the DNA level. This section introduces the four basic types of GA rodents, those created by: (i)

pronuclear microinjection, (ii) vector-mediated transgenesis (iii) homologous recombination in ES cells, (iv) gene editing nucleases, and (v) either chemically induced or spontaneous mutations. Detailed descriptions of the technologies used to create GAs have been published [32] [33] [34] [35]. Please also see the information provided by the International Society for Transgenic Technologies (ISTT) (http://www.transtechsociety.org/). Before selecting a gene-editing technique to create a genetically modified animal, it is important to check in an appropriate database such as those hosted by The Jackson Laboratories and the International Mouse Phenotyping Consortium (IMPC) as to whether a suitable animal model already exists (See Table 2). If a model is not available, then the most optimal method for generating the GA rodent must be selected.

2.1 Transgenesis by pronuclear microinjection

Transgenic mice were introduced in the early 1980s [36] [37] [38], and were the first *transgenic animals.* It is advisable to use the term 'transgenic' only for animals whose genomes have been altered by the random insertion of DNA[1] . Transgenic rodents are

---

[1] There are numerous terms used to describe genetic changes in animals. Genetically engineered mice (GEM) or genetically modified mice (GMM) are typically used to describe any type of genetic modification in the mouse. We use the term genetically altered (GA) rodent here to also include those carrying spontaneous or chemically-induced mutations. We use "line" instead of "strain" for GA rodents.

almost exclusively created by the pronuclear microinjection of foreign DNA fragments directly into one of the two pronuclei of one-cell embryos (zygote), a technique that is still widely used. In this process of *additive transgenesis*, the microinjected transgene randomly integrates into the genome as a single copy or as a concatemer with variable copy number. The mouse and rat models created with this system typically express or in the resultant concatemer, overexpress a transgene placed under the control of a tissue-specific, developmental-stage-specific, or ubiquitous promoter (along with other regulatory elements), all contained in the transgene DNA construct.

The recommended generic symbol for a transgenic insertion is Tg. The founder transgenic animals are hemizygous for the DNA segment and are designated Tg/0[2]. Each transgenic line generated via random integration creates a unique animal model and each putative founder must be developed independently. Establishing a transgenic line, in which the transgene is propagated by sexual reproduction, requires genotyping each generation to detect to which offspring the transgene was transmitted, unless the carriers have an obvious phenotype. Lines are normally kept by backcrossing transgenic carriers (hemizygous Tg/0) with wild-type animals from the inbred background strain and by selecting carriers at each generation. When viability and fertility are unaffected, a transgene may be maintained by keeping

---

[2] Transgenes are extra segments of DNA that have no corresponding "wild-type" sequence in the unmodified homologous chromosome in hemizygous animals, that is why it is recommended the use of "0" instead of "+" (typically used to denote wild-type alleles).

transgenic lines in the homozygous state. Traditionally, to distinguish between homozygous (Tg/Tg) and hemizygous (Tg/0) mice, the mouse of interest was crossed to a non-transgenic partner and the progeny were statistically analysed for Mendelian segregation of the transgene. A more modern technique uses quantitative real-time PCR (qPCR) and digital PCR to distinguish hemizygous from homozygous transgenic mice [39]. In order to achieve a pure genetic background (recommended), the transgene must be introduced into embryos derived from an inbred strain, such as FVB/N, which is widely used because its zygotes possess large and prominent male pronuclei, and the females are excellent breeders that produce large litters [40]. However, care should be taken when selecting the background strain and its intended research area. If the background utilised during production is inappropriate, a costly (time and animal numbers) process of backcrossing may be required (e. g., FVB/N is not appropriate for many behavioural studies).

A later improvement on the constructs used in the transgenesis approach was the introduction of inducible systems in which transgene expression can be turned on and off. Examples of this strategy are the Tet-on and Tet-off expression systems. In these systems, transcription of a given transgene is placed under the control of a tetracycline-controlled trans-activator protein, which can be regulated, both reversibly and quantitatively, by exposing the transgenic mice to either *Tetracycline* (Tc) or one of its derivatives, such as *Doxycycline* (Dox). Both Tet-on and Tet-off are binary systems that require the generation of double transgenic (*bigenic*) mice.

These mice carry both a responder construct consisting of a tetracycline response element- (TRE-) regulated transgene and an effector construct (tTA or rtTA) containing a tetracycline-controlled transactivator. This technology was developed by Bujard and colleagues at the University of Heidelberg [41] [42].

2.2 Vector mediated transgenesis

Alternative methods for transgenesis by random integration are based on vectors of different origin. Most important and very efficient are retroviral/lentiviral vectors [43]and transposons [44]. Also, pre-treated spermatozoa has been successfully used as vectors in combination with ICSI (intracytoplasmic sperm injection) [45]. Each technique has advantages and disadvantages and the corresponding principle of transgene integration may affect the quality of the resulting GA models. Viral vectors and transposons for instance integrate as a single copy, however multiple integrations, randomly distributed in the genome, are not uncommon. Major concerns exist regarding the impact of sperm-mediated gene transfer on the sperm genetic material, possibly induced by the pre-treatment of spermatozoa [46].

2.3 Targeted mutagenesis by homologous recombination using ES cells

Another important technology utilizes murine embryonic stem cell lines (ES cells). ES cells are undifferentiated, pluripotent, embryonic cells derived from the inner cell mass of pre-implantation blastocysts that can participate in forming the germ-cell lineage of chimeric mice, an indispensable step in generating founder mice carrying

the targeted mutation. Historically, the first ES cell lines were derived from embryos of the 129 family (129S2, 129P3, etc.), i.e. inbred strains originally bred for the isolation of embryonic carcinoma (EC) cells. Today ES cell lines are available from many mouse strains, and those of the C57BL/6N origin have become widespread and are often selected for trans-national projects (e.g., EUCOMM). In contrast, rat germline-competent ES cells have been developed much later, when compared to mice ES cells, [47] and their use is currently limited to a few specialised laboratories.

Chimeric mice resulting from the admixture of engineered ES cells with cells of the inner cell mass of a recipient blastocyst (less commonly 8-cell or morula stage embryos) can be identified on the basis of their dappled coat colour soon after birth, especially when the ES cells were derived from C57BL/6N (non-agouti *a/a*, general black appearance with yellow hair behind pinnae) and the recipient blastocyst was from either a wild-type (agouti *A/A*) or albino (Tyr^c/Tyr^c) strain. In these conditions, the chimeras exhibit a mixture of black and agouti or albino spots. Using coat colour as a reference, one can estimate the degree of chimerism, but a high level of chimerism does not necessarily parallel with a high rate of germline transmission. The majority of ES cell lines are XY, therefore, although chimeras can be from either sex, the number of males is usually unproportionally high within high chimeric litters and chimeric males are generally the only sex with germline transmission. In order to avoid mixed background lines, co-isogenic KO/KI lines should be generated by crossing the chimeras with wild-type mice from the same background as the ES cells.

For example, when C57BL/6-derived ES cells are injected into albino C57BL/6-*Tyr*^{c}/*Tyr*^{c} blastocysts, chimeric mice are easily identified by their white and black patches. These chimeras can then be crossed with albino C57BL/6 mice to test germline transmission, validated by the appearance of ES cell-derived black offspring [27] (note that the germline transmitted offspring will be heterozygous for the targeted allele as well as the mutant *Tyr*^{c} allele).

In cases where constitutive null alleles lead to complex phenotypes, viability may be affected, or have other drawbacks, conditional alleles may be used, allowing one to control the time and tissue where a gene is turned off, typically using the Cre/*lox*P system [48]. Production of conditional KOs requires two independent lines: one providing a source of Cre recombinase, an enzyme derived from bacteriophage P1, in the tissue under study, and another containing *lox*P (locus of X-ing over P1) sites flanking the DNA segment of interest that needs to be crossed to generate double mutant mice. The Cre enzyme cuts and recombines the '*floxed*' DNA at *lox*P sites. When the *lox*P sites are in the same orientation and on the same strand (in cis), the intervening stretch of DNA is excised. When two *lox*P sites are in the opposite orientation and on the same chromosome the intervening DNA segment is inverted. Finally, when the *lox*P sites are on two different chromosomes (in trans) the recombinase generates a reciprocal translocation. Other strategies for creating conditional mutants include the Flp-*FRT* and the Dre-*Rox* systems [49] [50].

The Cre transgene can be made inducible, adding more sophistication to the system. The tamoxifen-inducible Cre^{ERT2}, which can be activated in a spatio-temporal manner by administration of tamoxifen, is widely used [51]. However, tamoxifen treatment during early pregnancy can impair placental developments, resulting embryonic death and abortion [52]. Many Cre-expressing lines are being produced as KI mice that incorporate the Cre sequence directly into the gene of interest rather than by creating transgenic lines by pronuclear microinjection. The Cre-*lox*P strategy can also be used to regulate the expression of reporter genes. For example, the *lac*Z gene can be driven by a ubiquitous promoter (e.g. *Rosa 26*) with a floxed stop sequence, containing several terminator codons inserted between the promoter and the *lac*Z coding sequence. A cell or tissue specific Cre expression will result in a corresponding cell or tissue specific activation of the *lac*Z reporter gene [53]. Many available Cre expressing strains (Cre-deleters) have been added to The Jackson Laboratory Cre Resources database (http://www.creportal.org/).

2.4 Gene editing using nucleases

Over the last ten years, a number of new techniques have been developed for the production of targeted mutations using engineered nucleases. These techniques, briefly described below, provide ES cell-independent methods to create targeted mutations in laboratory mice, rats, and other species [54].

2.4.1 Zinc-finger nucleases and transcription activator-like effector nucleases

To make mutations using zinc-finger nucleases (ZFN), two complementary and sequence-specific multi-finger peptides containing the *FokI* nuclease domain must be designed. Each peptide is designed to recognize a specific DNA sequence spanning 9-18 base pairs (bp) on either side of a 5-6 bp sequence, which defines the targeted region. When injected into a pronucleus or cytoplasm of zygotes, the ZFN assemblies bind tightly, one on each strand, on both sides of the target site. The dimerized *FokI* endonuclease then creates double strand DNA breaks (DSBs) triggering cellular mechanisms to repair the damage. Damage is normally repaired by either homology-directed repair (HDR) or non-homologous end joining (NHEJ). HDR requires a homologous template to guide the repair and thus re-establishes the original sequence. NHEJ is much less precise and cause nucleotide deletions that lead to frame shifts that create potential loss-of-function or truncation mutations. Nonetheless, with ZNF technology, a homozygous KO mutation can be obtained in 4-5 months, much faster than with gene targeting in ES cells, which requires ~12 months. An important advantage of ZFN technology is that it is applicable to all strains of mice and rats, allowing for the production of mutations in different inbred backgrounds. Mice and rats carrying null alleles or sequence-specific modifications have already been produced using ZFN technology [55 56 57, 58]. Like ZFNs, transcription activator-like effector nuclease (TALEN) technology involves the combination of a nonspecific DNA endonuclease fused to a DNA-binding domain, but can be more easily engineered (compared to ZFN) to target a particular DNA sequence. Several

groups have used TALENs to modify endogenous genes in species including zebrafish, rat, mouse, pig, and cow [59] [60].

2.4.2 The CRISPR/Cas System

The CRISPR (clusters of regularly interspaced short palindromic repeats) -Cas system, commonly implemented as CRISPR/Cas9, is based on a primitive defence mechanism that allows bacteria and archaea to fight against infection from viruses, plasmids and phages [61] [62]. CRISPR-based guide RNAs (gRNAs) are designed to target a Cas endonuclease to cut DNA at the desired site through RNA-guided DNA cleavage.

The RNA-guided endonucleases can be engineered to cleave virtually any DNA sequence by appropriately designing the gRNA, for example to generate KO mice [63]. CRISPR/Cas technology has several advantages over ZFNs and TALENs. The main advantage is the ease of design and the flexibility of using a sequence-specific RNA interacting with the Cas enzyme instead of a complex sequence-specific protein (DNA-binding domain) fused to a nuclease. Also, mutations in multiple genes can be generated in a single step by injecting mice with multiple gRNAs that simultaneously target different genes [64]. Such multiplex gene editing has been successful in cells, as well as mouse and rat embryos [63] [65] [66] [67]. CRISPR/Cas9 has been used to create insertions, deletions, and point mutations. The system is highly flexible, fast, and efficient, and is revolutionizing genomic engineering in mammals [68] [69] [70] [71] [72] [73]. It allows making KO and KI lines in any genetic background. DNA can be electroporated (with size restrictions) [74] or injected into either the cytoplasm or pronuclei of 1-cell,

or 2-cell stage embryos [75], thus avoiding the use of ES cells and chimeras. However, as each engineered animal is unique, this technology requires extensive sequence analysis to characterize multiple putative founders to ensure the presence of the desired mutation and the absence of undesired on- and off-target mutations or unpredictable larger genome alterations [76] [77] [78], while also identifying mosaic founders (G0). Once identified, the selected founder should be bred with wild-type animals to evaluate transmission of the mutation to their offspring.

2.5. Spontaneous and chemically induced mutations

A list of GA rodent types is not complete without including both spontaneous and chemically induced mutations. Spontaneous mutations, generally identified through the observation of an abnormal phenotype, present several advantages. First and foremost, they are produced at virtually no cost and are generally freely available. Second, they usually have an obvious phenotype, as they are identified based on observation. Third, spontaneous mutations represent a great variety of molecular events, such as deletions, insertions, and point mutations, generating not only loss-of-function alleles but also hypomorphic and hypermorphic alleles. Finally, mutations arise in a variety of backgrounds including inbred strains and outbred stocks. Several spontaneous mutations have provided rodent models for human conditions. These include classical mutations such as, *nude* (*Foxn1*^{nu}), *scid* (*Prkdc*^{scid}), *hairless* (*Hr*^{hr}), *diabetes* (*Lepr*^{db}), *obese* (*Lep*^{ob}), and *X-linked muscular dystrophy*

(Dmd^{mdx}) in the mouse; and the mutations behind the Rowett nude ($Foxn1^{rnu}$), and Zucker diabetic fatty ($Lepr^{fa}$) models in the rat.

The discovery of the extraordinary virtues of the alkylating agent N-ethyl-N-nitroso urea (ENU) as a mutagen was a milestone in the history of mouse genetics. Researchers using ENU have generated and propagated numerous mutant alleles for protein-coding genes, thus establishing a precious tool for genome annotation. Because ENU typically creates point mutations, it has been widely used in forward genetic screens. The major drawback of ENU-induced mutagenesis is that it creates random mutations rather than targeted mutations. Several projects have been undertaken to systematically and extensively phenotype the offspring of ENU-mutagenized males. Large ENU mutagenesis programmes have been conducted in Germany, England, and the USA [79].

2.6. Quality assurance and exchange of GA-rodents

2.6.1. What to ensure after (in-house) generation or upon arrival?

The possibility of crossing different GA lines combined with the increasing complexity of targeting approaches has greatly increased the number of available GA models. The need to cross different GA lines together for a particular study generates additional complexity, especially at the genetic background level. Many mutants have been and are still generated on a hybrid genetic background. Therefore, it is essential

to keep adequate records of detailed information for all genetically modified strains. This information must be transferred with the strain to all collaborators and users. The most important information includes the correct strain name, a complete description of the mutation, the background of the animals, a genotyping protocol, and observed phenotypic changes. Together, these provide the minimum information for the recommended 'rodent-passport', and several forms have been designed for cataloguing this information. We recommend the data sheet developed by the FELASA Working group on the refinement of methods for genotyping genetically-modified rodents [80].

Every mutant strain name must provide precise information on the affected gene, the type of mutation, and the genetic background. For this purpose, a standardized nomenclature of strains, genes, and mutations has been established by the International Committee on Standardized Genetic Nomenclature for Mice and the Rat Genome and Nomenclature Committee (see Section 4). For in-house generated strains, one must provide a specific Institute for Laboratory Animal Research Laboratory (ILAR) Code Registration for the laboratory where the mutant originated. An overview on the importance of nomenclature can be found in the 'FELASA guidelines for the production and nomenclature of transgenic rodents' [81]. A name designed according to the international nomenclature rules is the only means to unambiguously distinguish strains from each other. This is important when the same strain is held in different facilities around the world and/or they are listed in

archives and databases. Further, it is imperative that strains be properly described in publications using a universal nomenclature. Without a common nomenclature, it becomes impossible to accurately communicate scientific results. Vague or incomplete names create errors rendering experiments irreproducible.

2.6.2. Description of the genetic modification

A complete description of the genetic modification and/or transgene used should always be included when describing a GA model. A schematic outlining the transgene structure used and containing precise information about the gene regulatory and coding regions is valuable. For transgenic mice, the insertion site is not typically known; however, if known, the sequences flanking the integration site should be provided. Random integration sites can be identified by ligation-mediated PCR, followed by cloning and sequencing of the flanking genomic sequences [82]. For gene traps, the locus of the insertion and description of the targeted locus is usually identified. This information is available from ES cell providers, such as the International Gene Trap Consortium (http://www.genetrap.org). Strains generated by gene targeting should have a detailed description, at single-nucleotide resolution, of the targeted locus that includes the positions of exons, introns, and possible sites for *lox*P and *Frt*-sequences. Also include the information needed for confirming the targeted allele by Southern blotting, the ES cells used for targeting, the PCR primer pair sequences for routine genotyping; and the site and type of any putative antibiotic selection and/or reporter gene inserted into the locus. This information should also be provided electronically to facilitate analysis of the modified locus. For

published strains, a reference to the publication originally describing the mouse model must be included.

2.6.3. Quality assurance for genotyping samples

Genotyping GA mice is a routine procedure. The FELASA Working group on the refinement of methods for genotyping genetically-modified rodents has created a detailed report on current methodologies for sample collection and genotyping [80]. Some important points are highlighted below.

Rodent genotyping protocols should be specific, simple, easy, and robust, and avoid animal harm. The method of sample collection and genotyping must not stress the animals. Genotyping is the only reliable way to maintain a colony over time and to share bona fide mouse strains between scientific collaborators. One important prerequisite is that a reliable animal identification system is used, as no genotype information is valid without an unequivocal way to link it to the individual animal at all times. Mice and rats must be marked with a well-defined identification code that, together with genotype information, should be passed to the recipient of the mice at a different location. Once selected, the most appropriate genotyping protocol should be recorded along with the strain information, so that it may be faithfully reproduced in the new facility. Routine genotyping of GA lines is preferably performed using PCR, to identify WT, heterozygous, and homozygous animals. Information regarding primer sequences and cycling conditions should also be recorded and provided with the strain. Sometimes it is possible to identify mutants by phenotype alone. However,

distinguishing mutant and WT littermates by macroscopic appearance is ill-advised: phenotype can vary between carriers, especially between generations and in different genetic backgrounds.

2.6.4. The challenges of using CRISPR/Cas9 systems

CRISPR technology allows the generation of double and triple mutants within a few months, significantly reducing time and costs, particularly by eliminating problems created by background strain differences. Further, CRISPR/Cas9 microinjection into embryos produces a large proportion of founder mice that are homozygous for the edited alleles, thus allowing rapid phenotypic analysis without additional breeding. However, from a genetic viewpoint, this very powerful technology has some drawbacks. First, the majority of gRNA-targeted Cas9-induced DSBs are repaired by error-prone NHEJ, so unintended modifications and drastically reduced efficiency can occur when the goal is to induce precise mutations into a locus via HDR (see http://dx.doi.org/10.1101/393231). Second, microinjection of Cas9 mRNA and gRNA into single cell zygotes often causes genetic mosaicism in founder animals (although this risk can be reduced using Cas9 protein instead of mRNA/RNP complexes). Cas9:gRNA is delivered to zygotes during the period of active DNA replication, thus, editing may occur either before or after a particular locus has been replicated, with the latter potentially resulting in mosaicism. Additionally, due to the persistence of Cas9:gRNA complexes, editing may occur only in one blastomere after the first cell division.. In some cases, mosaicism can reach up to 80% [83], and constructs targeting early replicating regions would be more likely to generate

genetic mosaics, as there would be four copies available for editing if Cas9 acts upon replicated DNA.

3. Origin and consequences of genetic variation

A serious challenge facing rodent animal facilities is keeping inbred strains genetically pure and GA lines on a defined background. Changes in the genetic constitution of inbred strains can be produced by (i) contamination by an accidental outcross or multiple outcrosses, and (ii) genetic drift due to residual heterozygosity or fixation of *de novo* spontaneous mutations.

3.1 Genetic contamination

The accidental mating of individuals from one inbred strain with animals of another origin is by far the most important source of genetic profile alteration in inbred strains. Genetic contamination of this type, which always results in a sudden and massive exchange of alleles, is more likely between strains that have similar coat colour [*i.e.*, albino (Tyr^c/Tyr^c), agouti (*A/A*), or non-agouti (*a/a*)]. Where lines have the same coat colour alleles, extra care must be taken when housing them in close proximity of each other. A well-known example of genetic contamination is the former C57BL/Ks strain (now C57BLKS) that derives from strain C57BL/6, but was contaminated with up to 30% DBA/2 [84]. In the rat, the WKY strain and the Dahl salt-sensitive (SS/Jr) strain have been reported as genetically contaminated and having genetic heterogeneity between breeding facilities [85 86].

3. 2 Spontaneous mutations and polymorphisms

Spontaneous mutations are a source of uncontrolled genetic variation that is often impossible to detect by simple phenotypic observation or routine GeMo. Estimated rates of spontaneous mutation in mammals range from 10^{-5} to 10^{-8} per locus per gamete, depending on whether being assessed based on breeding or sequencing data. If the mutation rate is 0.5–3 x 10^{-8} per bp per generation, then approximately one protein-coding mutation per generation is expected to arise through genetic drift [87].

Genetic polymorphism is the presence of alternative DNA sequences (alleles) at a locus among individuals, groups, or populations, at a frequency > 1%. In the laboratory rat and mouse, analysis of these genetic variations has evolved with the need for genetic markers in linkage studies. An enormous number of genetic markers, polymorphic between inbred strains, has been instrumental for developing genetic maps and identifying genes by positional cloning, but these markers can also be used for GeMo and background characterization [1]. Two types of genetic markers are commonly used in association studies and genetic quality control: microsatellites and single nucleotide polymorphisms (SNPs) (see Section 5.1).

3.3 Genetic drift and the generation of substrains

While permanent inbreeding effectively eliminates a proportion of new mutant alleles, another undetected fraction may become progressively fixed in the homozygous state, replacing the original allele, a process known as *genetic drift*.

Genetic drift contributes inexorably to strain divergence and the generation of substrains when the same strain is propagated independently in different places [88]. Examples of mouse substrains are abundant, for example there are ~10 documented BALB/c substrains and ~15 C57BL/6 substrains including the J and N substrains from The Jackson Laboratory (Jax) and the National Institutes of Health (NIH), respectively [89] [90]. The 129 family of inbred substrains is very unusual for its high level of divergence, including different coat colours. For example, 129X1/SvJ and 129P3 strains are albino (or chinchilla), whereas 129S1, 129S4, 129S6, 129S7 (*Still* group) are agouti [91] (see http://www.informatics.jax.org/mgihome/nomen/strain_129.shtml). In the same way, many rat inbred strains present at least two substrains, for example SHR has at least four substrains (including SHR/Ola and SHR/NCrl), and WKY and F344 have at least three substrains each. Substrain variability has been confirmed by sequencing analysis for these rat substrains [92], with WKY showing the highest degree of substrain variation (this is in part due to the supply of the model prior to the prescribed 20 generation inbreeding requirement).

The insidious and unavoidable occurrence of new mutations in strains justifies the recommendation in the Guidelines for Nomenclature of Mouse and Rat Strains that inbreeding should never be relaxed. Inbreeding is inefficient in preventing mutations, but helps eliminate a substantial proportion of new mutant alleles, thus preserving the genetic profile of a given strain. Similarly, the same international committee on

nomenclature has stated that two strains with the same origin, but separated in different colonies for 20 or more generations (for example, 12 in laboratory A and 10 in laboratory B) should be considered two different substrains and designated appropriately. The Institute for Laboratory Animal Research (ILAR) maintains the International Laboratory Code Registry (http://dels.nas.edu/global/ilar/Lab-Codes). Each lab code contains one to five letters and identifies the institute, laboratory, or investigator that produced and/or maintains a particular strain.

3.4 Undesirable passenger mutations

Mutations that are hidden in the genomes of substrains or GA lines and can affect the outcome of an experiment are sometimes referred to as *passenger mutations* [93]. There are many examples in the literature where substrains originating from the same inbred strain have acquired new phenotypes as a consequence of genetic drift [94]. For example, mice of the C57BL/6JOlaHsd substrain are homozygous for a deletion of the α-synuclein (*Snca*) and multimerin (*Mnrn1*) genes [95 96]. This deletion has modest phenotypic effects but might interfere in an unpredictable manner with other KO mutations [97]. Likewise, some spontaneous mutations differentially segregate in C57BL/6J and C57BL/6N, the most common substrains of C57BL/6, separated in 1951. These include a retinal degeneration mutation in the *Crb1* gene ($Crb1^{rd8}$) and a non-synonymous SNP in the *Cyfip2* gene, present only in the N substrain, and a deletion in the *Nnt* gene, present only in the J substrain [98 99 100]. A comprehensive comparative phenotypic and genomic analysis of C57BL/6J and

C57BL/6N was recently published [101]. Similarly, C3H/HeJ (Jax) and C3H/HeN (NIH) mice may react very differently when infected with Gram-negative bacteria due to a homozygous mutation in the *Tlr4* locus (encoding for a *Toll*-like receptor) in C3H/HeJ mice [102]. Berghe and colleagues recently reported that passenger mutations are also common in most GA lines derived from 129 ES cells, and that these mutations persist even after the creation of fully congenic strains [103]. This is not trivial; Berghe *et al.* estimated that close to 1,000 protein-coding genes could be aberrantly expressed in the 129-derived chromosomal segments that are still segregating in these congenic lines. This finding emphasizes the need for properly chosen control animals to identify phenotypes due to background mutations or the combination of background mutations and the genetic modification of interest, rather than the modification itself.

4. Importance of using standard nomenclature

Naming and describing inbred strains with standard nomenclature is critically important [81] [104]. Rules guiding nomenclature were established by the International Committee on Standardized Genetic Nomenclature for Mice and Rats and are continuously updated. These rules, last revised in January 2016, are described on the MGI webpage under '*Guidelines for Nomenclature of Mouse and Rat Strains*' (http://www.informatics.jax.org/mgihome/nomen/strains.shtml). A helpful and visual Mouse Nomenclature Quick Guide is available at https://www.jax.org/jax-

mice-and-services/customer-support/technical-support/genetics-and-nomenclature#

Some of the essentials of standard nomenclature follow.

(i) An inbred strain should be designated by a unique, brief symbol consisting of uppercase, roman letters, or a combination of letters and numbers beginning with a letter (e.g., C57BL). However, some pre-existing strains, like 129 strains, do not follow this convention.

(ii) Substrains are given the symbol of the original strain, followed by a forward slash and a substrain designation, usually the Laboratory Code of the individual or laboratory originating the strain, for example A/He substrain from Walter Heston.

(iii) Congenic strains are designated by a symbol consisting of three parts. The full or abbreviated symbol of the recipient strain is separated by a period from an abbreviated symbol of the donor strain, which may or may not be its immediate source in constructing the congenic strain. In cases where the chromosome on which the mutation arouse is unknown (e.g., the donor is not inbred, or is complex, or an F1 hybrid), the symbol Cg should be used to denote this complex genetic origin. A hyphen then separates the strain name from the symbol (in italics) of the differential allele(s) introgressed from the donor strain, for example NOD.CB17-$Prkdc^{scid}$. If the recipient strain is of unknown or complex background it can be stated as STOCK.

(iv) F1 hybrids are designated using the full names or the abbreviations of the two parental strains (maternal strain listed first), followed by F1. For example, (C57BL/6

x DBA/2)F1 or abbreviated to B6D2F1. Keep in mind that the reciprocal F1 hybrids are not genetically identical due to their different sex chromosomes and mitochondrial DNA (in this case D2B6F1).

(v) In the case of mixed inbred strains (one of which could be the ES cell line used for the targeting), they can be designated using upper case abbreviations for the strains, separated by semicolons. For example, B6;129-*Lrrc8a*^{tm1Geha}.

(vi) For outbred stocks, the common strain root is preceded by the Laboratory Code of the institution holding the stock. For example, Tac:ICR is the ICR outbred stock maintained by Taconic Farms, Inc.

Finally, remember that mouse and rat gene symbols are italicized and begin with an uppercase letter followed by all lowercase letters (human gene symbols are also italicized but are written in all uppercase letters). When the causative gene is not known, mutation symbols should be written according to the inheritance mode of the mutation: i) recessive mutation, all with lowercase letters, ii) dominant or semi-dominant mutation, begin with an uppercase letter followed by all lowercase letters.

5. Genetic quality control programmes

The current gold standard for genetic quality control of laboratory rodents depends on polymorphic *genetic markers* to distinguish between different genetic backgrounds. Genetic markers are specific DNA sequences with a known location on

a chromosome, and are essential tools for genetic quality control. Genetic quality control is essential to determine the genetic composition of an animal and to screen for uniformity and authenticity of a strain[3], to detect genetic drift, and genetic contamination and to monitor the progress of breeding programmes and to select future breeders.

5.1 Marker Systems

Many polymorphisms have been described in the mouse and rat; however, only microsatellites and SNPs are used as genetic markers in current QA programs. Historically, biochemical markers, especially enzyme polymorphisms, were used extensively for GeMo of inbred strains. Immunological markers, especially those of the Major Histocompatibility Complex (MHC), called the H2 complex in mice and the RT1 complex in rats, were also used to verify genetic authenticity. The advent of DNA profiling/fingerprinting methods introduced restriction fragment length polymorphism (RFLP) analysis using probes directed to minisatellite DNA sequences. RFLP technology was quickly adapted for GeMo of inbred strains [105], but has been largely replaced with less cumbersome, modern methods. It is still early to assess whether whole-exome or whole-genome sequencing will be useful for general QA purposes. Currently, whole-exome sequencing provides a robust method for discovering hereditary factors controlling rare Mendelian disorders in humans, as

[3] Outbred colonies can not be tested for authenticity. Instead, the colony is screened for its level of genetic heterogeneity.

well as new mutations previously mapped through positional cloning in mice and rats [87], and could be useful for the characterization of substrains.

5.1.1 Microsatellite markers

Microsatellite markers, also known as Simple Sequence Length Polymorphisms (SSLPs) or Short Tandem Repeats (STRs), are still used in modern GeMo programs because they are inexpensive and easy to type [106 107 108]. Animals are genotyped by analysing PCR-products amplified from short, tandemly arranged, repeating DNA sequences. These repeats are typically 2-6 bp long, and are repeated a few to dozens of times creating allelic diversity among stains. Genomic DNA primers are designed to unique sequences flanking the repeats. The PCR products, typically around 100-300 bp in size, are analysed using agarose or polyacrylamide gel electrophoresis. There are an enormous number of microsatellite loci in the mouse and rat genomes ($\sim 10^5$), and it is generally possible to identify a set of markers whose amplification products define a strain-specific pattern. Routine analysis of DNA samples with carefully selected microsatellite markers will confirm both the authenticity and isogeneity of an inbred strain. Because microsatellite markers are multiallelic markers, each can identify multiple alleles from different strains, that can be differentiated by their individual amplification product sizes (in some cases up to 8 different allele sizes from one marker). The use of fluorescently labelled primers to amplify microsatellite loci combined with capillary electrophoresis provide an even faster and more automated means for genetic monitoring [108]. With this method, the resulting PCR products can be distinguished from one another by both their size and

the fluorescent dye associated with them, which aids in multiplexing the PCR reactions.

The MGI webpage has comprehensive SSLP information, including primer sequences and size variations in bp for several inbred mouse strains (http://www.informatics.jax.org/marker). A collection of mapped, highly polymorphic, SSLP markers for inbred laboratory rat strains is available in The National BioResource Project – Rat database and is linked to the Map Report of the Rat Genome Database (RGD) (http://rgd.mcw.edu).

5.1.2 Single Nucleotide Polymorphisms (SNPs)

SNP genotyping is an alternative to microsatellites that is now widely used for GeMo. SNP genotyping is inexpensive and can be performed in most research institutions or outsourced. SNPs are the most common genetic variation and exist in both coding and non-coding regions. Almost all SNPs are bi-allelic, presenting one of only two possible nucleotides (e.g., homozygous G/G or T/T), or both (e.g., heterozygous G/T) in an individual. Petkov and co-workers from The Jackson Laboratory have described the allelic distribution of 235 SNPs in 48 mouse strains and selected a panel of 28 SNPs sufficient to characterize the majority of the ~300 inbred, wild-derived, congenic, consomic, and recombinant inbred strains maintained at The Jackson Laboratory [109]. This set of markers, encompassing all mouse chromosomes, is an excellent tool for detecting genetic contamination in mouse facilities. The same laboratory developed a set of 1,638 informative SNPs selected from publicly

available databases, and tested them on 102 inbred strains using Amplifluor genotyping [110]. Several publications have reported useful SNPs for the rat. Zimdahl and colleagues described a map with >12,000 gene-based SNPs from transcribed regions [111]. In another study, 485 SNPs were identified in 36 commonly used inbred rat strains [112]. More recently, the STAR (RATS backward) consortium reported a set of 20,000 SNPs across 167 inbred rat strains [113]. SNP genotyping assays are currently based on allele-specific PCR (including KASPar fluorescent technology) [114], real-time PCR (TaqMan®), direct sequencing, and DNA arrays [115].

5.2 Genetic monitoring (GeMo) of inbred strains and outbred stocks

Most GeMo techniques used currently are based on microsatellites or SNPs. However, GeMo should not rely solely on molecular techniques, but should take a broader view that includes phenotypic parameters such as coat colour, behaviour, and breeding performance. Commercial breeders are extremely sensitized to the risk of genetic contamination and regularly monitor their strains for genetic contamination, but not necessarily genetic drift, by using different sets of SNPs to monitor their nucleus colonies. The Jackson Laboratory incorporated a unique, patented, Genetic Stability Program [116] designed to effectively limit cumulative genetic drift by rebuilding their foundation stocks from pedigreed, cryopreserved embryos every five generations. For example, starting in 2005, they began selling only C57BL/6J mice derived from two chosen mice (Adam and Eve mice) through hundreds of frozen embryos of the duo's grandchildren, enough to last for 25-30 years [117]. The complete genome of the

“Eve” female mouse was recently published [118]. It should be noted that when recovering strains from frozen stocks good GeMo should be carried to assure oneself genetic contamination or wrong genotypes were not present prior to freezing. Most other vendors also have backup archives of their stocks cryopreserved in an embryo bank, allowing the rapid development of a fresh nucleus colony when necessary. The International Council for Laboratory Animal Science (ICLAS) is now emphasizing GeMo programs and helping academic institutions develop and implement them to improve overall QA for mouse and rat models. Current ICLAS recommendations were recently reviewed in Fahey et al. [119].

It is important to observe animal phenotypes when monitoring breeding colonies. Most phenotypic traits are polygenic, resulting from the effects of multiple genes, some of which are pleiotropic and influence more than one trait. The phenotype can also be modified by environmental factors, such as the conditions in which the animal was born and raised. Therefore, a robust monitoring plan of quantitative traits should be based on measurements of an appropriate number of animals. Monitoring should include observation of spontaneous (innate) behaviour, which differs between inbred strains of laboratory rodents; maternal care and rearing behaviour, which can be assessed during routine breeding work; and obvious phenotypes such as coat colour should always be considered. Detailed information on the genetics of rodent pigment variation have been summarized in textbooks [120, 121] and genome databases (**Table 2**).

Robust GeMo programs also monitor reproduction parameters including the general fertility and health of a strain: the average number of animals born and weaned per litter at any one time period in standard environmental conditions, can reveal substantial genetic changes. The monitoring should also be performed on a representative random sample of pairings to avoid any inadvertent bias towards poor breeding pairs. Sudden increases in litter sizes or elevation of the breeding index [122] in an inbred strain strongly indicates possible genetic contamination. Identifying strain-specific pathologies is also important for discovering possible genetic contamination and genetic drift. Some examples of strain-specific pathologies in mice are teratocarcinomas in 129, thymic lymphomas in AKR, and Type 1 diabetes in the Non-obese Diabetic (NOD) strain (a model for Type 1 IDDM in man) [123]. Examples in rat inbred strains are Type 2 diabetes in the Zucker diabetic fatty rat (ZDF) and hypertension in the spontaneously hypertensive rat (SHR) [85].

For outbred stocks, GeMo helps preserve the genetic heterogeneity and allele pool of a colony. This complex process requires analysing a large number of animals and comparing this data with historical data documenting the alleles present, their frequency, and the level of heterozygosity in that particular colony. In some cases, the results can reveal a loss of genetic variability resulting in a colony with very low heterogeneity. The degree of genetic heterogeneity in outbred colonies depends on their history. Low heterogeneity can result from poor selection of future breeding

stock, deviation from approved (rotational) breeding systems or the bottleneck effect caused by a small breeding pool, as is common when a small group of breeders is imported or being used to rederive a colony. In contrast, high heterogeneity can result from a recent outcross. In general, outbred stocks are characterized by measuring phenotypic traits and calculating the corresponding mean and standard deviations. Essentially, genetic control of outbred stocks is directed at avoiding inbreeding and stabilizing genetic diversity over many generations.

5.2.1 GeMo of inbred mice and rats bred in-house.

The best recommendation here is to purchase animals from reliable vendors and replace the breeding stock with animals from the same vendor after 10 generations, rather than to maintain independent colonies of classical inbred strains. As an additional benefit, using animals from the same vendor prevents the formation of substrains harbouring potential mutations. Nevertheless, in-house colonies should always be tested with a small set of informative microsatellite markers or SNPs to confirm integrity.

Using a small panel of microsatellites (SSLPs)

Microsatellites can be used to verify that the animals in an inbred colony are essentially pure, with no traces of genetic contamination. This is especially important in facilities that maintain strains with the same coat colour in the same room, a particularly dangerous practice especially when not using individually ventilated cage (IVC) systems.

Microsatellite testing can normally be performed in-house. The number of markers to use for testing has not been standardized: each situation and facility differs in how many and which strains are kept. Nonetheless, a panel of 40 polymorphic SSLPs, evenly distributed across the 19 autosomes, will rule out recent genetic contamination, if the markers can distinguish among the strains being analysed. **Table 3** includes a small set of mouse SSLPs that could be used to authenticate some classical inbred strains.

SSLP genotyping is performed using standard PCR. Primers may be ordered from any reliable source. The quality and quantity of the DNA template obtained from tail clips or ear punches (also clipping the distal phalange of only one toe in mice under 7 days of age, or as approved by the ethical committee) should be assessed before performing the PCR reaction. Control PCR reactions using DNA templates from authenticated samples from each strain should be included to provide a precise size standard for the expected alleles. It is important to compare the band sizes to this standard rather than to estimate band sizes solely compared to the DNA ladder. Following amplification, PCR products are analysed on a 4% agarose gel. To distinguish among alleles, amplification products should differ by >4 bp. A simple SSLP amplification protocol is included in **Supplementary Material**. The cost of SSLP genotyping is very low for facilities with access to thermocyclers, gel electrophoresis equipment, and imaging systems. Reagent costs should not exceed

US $1.00/reaction, and the reagents are stable for years when properly stored. Alternatively, some institutional core facilities offer SSLP genotyping at reasonable prices.

Interpreting SSLP data is straightforward. Because inbred animals are isogenic and homozygous, they will present only one band in the gel, representing a single allele, when genotyped for a particular SSLP. The presence of any heterozygosity, indicated by two bands, or bands that do not coincide with those of the strain control DNA, should be considered as indicating potential strain contamination (**Figure 2**). It is important to note that the sizes reported in databases are not always accurate, and that differences in allele size from closely related substrains are possible. Mutations in SSLP loci are rare but possible (and most likely will not produce a phenotype). Using additional markers flanking the suspect SSLP should resolve the issue and help differentiate contamination from a *de novo* mutation affecting the SSLP. In the latter case, replacing the breeding colony is not required.

How frequently colony strain identity should be evaluated depends on the size of the colony, the generation interval, etc. Generally, testing once every two years is reasonable for a facility maintaining a small number of colonies well-separated in terms of coat colour, and with low numbers of importations. When a new inbred strain that is not available from other sources (vendors or repositories) has been developed from scratch, genetic QA should be much more comprehensive, starting

with the characterization of a genetic marker profile for the strain. Again, there are no standard rules; however, the more DNA (SSLPs and SNPs), immunological, and phenotypic markers, the better.

Using a small panel of SNPs

SNPs are now preferred choice for GeMo for most commercial suppliers of laboratory mice and rats. For GeMo purposes only, 40 polymorphic SNPs, evenly distributed across the chromosomes is a reasonable number for detecting recent genetic contamination (this suggestion should be modified dependent on the conditions or risks in each facility). SNP genotyping is currently available on different platforms that vary in cost and automation capabilities. Allele-specific PCR is probably the most convenient and inexpensive for minimally equipped laboratories [124]; however, it offers no advantages over SSLP genotyping. Nevertheless, this technique can be used to differentiate between substrains if informative SNPs are used. Kompetitive Allele Specific PCR (KASP), a variation on allele-specific PCR uses allele-specific oligo extension and fluorescence resonance energy transfer [110], has the advantage that it can be automated using 96- or 384-well plates and pipetting robots for the PCR reactions, and plate readers, equipped with appropriate software, for the analysis (**Figure 3**). In addition, hundreds of individual SNP KASP assays for mice and rats are commercially available (e.g., LGC in the UK). This allows customizing the numbers and identities of SNPs for each situation, and avoid using a fixed set of markers that may include non-informative SNPs for a particular application, which cannot be avoided when using SNPs arrays. Another option, real-time PCR (TaqMan®)

technology, uses specific primers coupled with a sequence-specific, fluorescent TaqMan probe, is effective and easy to automate; however, the cost per individual assay is expensive compared with KASP assays, and requires a more costly real-time thermocycler.

Finally, microarray-based SNP genotyping [115], is not typically used for small scale, in-house GeMo, but may be an option for vendors of inbred mice. When using or requesting microarray genotyping services, be aware that only a percentage of the SNPs will be polymorphic between the strains under analysis (e.g., ~40% for some classical inbred strain combinations). High-density microarrays developed for gene mapping purposes could also be used to create a complete SNP profile characterization for a new or poorly described inbred strain. For example, the Mouse Diversity Genotyping Array [125] [126] is a high-density mouse genotyping microarray (Affymetrix) that simultaneously assays over 600,000 SNPs. The Mouse Universal Genotyping Array (MEGA) in its MiniMUGA format has 11,000 SNPs, the MegaMUGA has 78,000 SNPs, and the GigaMUGA provides more than 143,000 SNPs. Both of these were built on the Illumina Infinium platform. The GigaMUGA array also includes non-SNP probes intended for exploring copy number variations (CNV). The majority of these SNPs are distributed throughout the mouse genome and were selected to be informative in most mouse strains (http://genomics.neogen.com/en/mouse-universal-genotyping-array).

As mentioned, information regarding which alleles (C, G, A, or T) to expect for a particular SNP/strain combination, and their genomic location are available for hundreds of thousands of SNPs and for the common mouse and rat inbred strains in easily accessed databases and genome browsers. For example, the Mouse Phenome Database (MPD), The Mouse Genome Informatics (MGI) site, the Sanger Institute's Mouse Genomes Project, the Rat Genome Database (RGD), and the Ensembl genome browser (**Table 2**).

5.2.2 Discrimination of substrains

The consensus is that if an inbred colony has been genetically isolated for more than 20 generations, it should be considered a substrain, regardless of whether the strain has been confirmed to be genetically different from the parental strain. SSLPs are not recommended for identification of substrains, because there are insufficient numbers of informative markers to compare most of the common substrains. To characterize a novel substrain, it is necessary to use a large set of SNPs. For example, a pairwise comparison of sister strains using the MegaMUGA array showed that the number of polymorphic SNPs is 154 between C57BL/6J and C57BL/6N, 134 between BALB/cJ and BALB/cByJ, and 827 between C3H/HeJ and C3H/HeN [127]. However, only complete exome sequencing, which is becoming more affordable, will provide complete information on mutations that might have occurred in protein coding genes that could influence a specific phenotype. Nevertheless, if the goal is merely to identify the classical substrain a colony or an animal is associated with, then a small number of SNPs can be selected, based on the information available in the SNP

databases. As an example, different SNP panels have been proposed to differentiate C57BL/6J from C57BL/6N [89] [128] [90]. Similar levels of SNP variability were described for several substrains of rats, like F344, SHR, and WKY [92].

5.2.3 GeMo of outbred colonies

GeMo of outbred stocks is much more complex, because these animals are not genetically uniform. Outbred colonies are essentially a group of closely related animals, with shared ancestors and group identity, but that exhibit some level of genetic heterozygosity. Since outbred colonies form a population rather than a strain, it is difficult to establish a standard GeMo programme with only a few genetic markers. However, with an adequate number of SNPs or SSLPs, allele frequencies within the population could indicate the identity of the stock [17]. One of the main problems of in-house outbred stocks is that they are often maintained with a very small number of animals in the breeding colony, causing a reduction in the number of alleles represented in the population. This may impact genetic drift and increase the inbreeding coefficient. Such colonies are neither truly outbred nor inbred. Although SSLPs or SNPs can be used to estimate the level of heterozygosity within the colony, if it is not possible to keep an appropriate number of breeders, it is better to purchase outbred rodents from vendors that maintain a very large colony and use recommended breeding schemes to reduce inbreeding.

5.3 Background characterization (BC) for GA and mutant lines

The explosion in the number of GA lines is exacerbating the problem of undefined "mixed backgrounds" in experimental rodents. This is particularly worrisome for inducible and conditional models that require the crossing of two independent lines (e.g., Cre-expressing lines crossed with floxed lines). Given that genetic background influences phenotype, especially through the influence of modifier genes; mutations, both spontaneous and induced, transgenes, and targeted alleles that are introgressed into a new background may not exhibit the expected phenotype [129] [130]. One of the first cases reporting this phenomenon involved the classical diabetes ($Lepr^{db}$) mutation that presented transient diabetes in a C57BL/6 background but overt diabetes in C57BLKS [131]. Other examples include background effects on survival rate in *Egfr* (epidermal growth factor receptor) KO mice [132], effects on tumour incidence and spectrum in *Trp53* and *Pten* KO mice [133] [134], and effects on acetaminophen-induced liver injury in *Jnk2* KO mice [135], to name only a few. There are also examples from rat models, like the influence of genetic background on prostate tumorigenesis in Pb-SV40 transgenic rats [136]. For this reason, every GA line should be characterized in terms of their genetic background. Moreover, the knowledge of the genetic background of a mutation is also important for the selection of the corresponding control animals [135].

Genetic markers evenly distributed and covering the entire genome can be used in a genome scan to estimate the percentages of genome coming from different inbred

origins. This process of background characterization (BC) is provided by some commercial enterprises and institutional core facilities. A typical BC employs polymorphic markers to distinguish between the suspected inbred strains. In most mouse cases, these strains are C57BL/6 and 129 substrains because, historically, the ES cells used for the development of KO and KI mice through homologous recombination (Section 2.2) were derived exclusively from 129 substrains [91], whereas WT C57BL/6 females were typically used to prove germline transmission from the chimeras. Without subsequent backcrosses, this scheme resulted in a B6;129 mixed background. However, the availability of ES cell lines derived from other strains (particularly from C57BL/6) and the arrival of genome editing techniques (Section 2.3) that allow direct production of targeted alterations in any mouse or rat strain [137] is slowly changing this scenario.

Again, markers for this type of background characterization have not been standardized, but a set of 100-150 SNPs or 100-120 SSLPs, polymorphic between the suspected strains should suffice. In cases where no strain information is available, the best option is to use a set of 100-120 SSLPs evenly distributed across the genome, and to include DNA controls from a set of commonly used inbred strains. A very important part of marker selection is determining how the GA line was developed and subsequently crossed, etc. The availability of strain-specific historical information is very important, and we strongly encourage the use of a rodent-passport (Section 2.5.1) containing information on GA animals [138]. Once this

information is known, a set of polymorphic markers can be selected to estimate the percentages of each genetic background.

In the case of classical transgenic mice, it is important to know the strain that was used for the pronuclear injection, plus the crosses made to develop the line (including ID of the founder). A common problem with transgenic mice is the use of hybrid F1 embryos for the injection of transgenes, followed by a cross of founders with randomly selected WT mice. This results in a mixed background line where each mouse has a different combination of the parental genomes present in the injected hybrid F1 embryo (e.g., D2B6F1). For the mutagenesis using ES cells, it is essential to know the origin of the ES cell line used for the targeting plus the preliminary crosses done for the germline transmission test and the establishment of the line (if known, number of backcrosses, etc.). If the ES cell line belongs to a different strain than the one selected to cross with the chimeras, the resulting KO/KI line may have a mixed background.

Bigenic lines carrying two independent transgenes, two targeted genes, or a combination of both, are also a concern. For example, the Tet-on (or Tet-off) inducible system requires the generation of double transgenic lines carrying the responder and the transactivator constructs in the same animal (Section 2.1). Likewise, conditional systems such as Cre-*Lox*P require double transgenic lines that carry both the Cre transgene (or KI locus) and the floxed targeted gene. In both cases,

it is very common to see mouse cohorts with mixed backgrounds because the original single-gene lines (e.g., the Cre-expressing line and the floxed line) were from different backgrounds or outbred stocks.

In any case, the problem of mixed background can be circumvented altogether by (i) injecting transgenes or nucleases (Cas9-sgRNA) into inbred embryos from the strain of choice; (ii) modifying the gene of interest in ES cells from the preferred background strain (e.g., using C57BL/6 ES cells); and (iii) crossing chimeras and KO/KI founders with mice of the same strain as the ES cells used for the targeting. Finally, if the GA has already been developed or acquired from a collaborator or repository, a background characterization should be performed, and if needed, a fully congenic strain should be developed, either by classical or marker-assisted backcrossing. Periodic backcrossing of a congenic strain to the background strain also minimises divergence and keeps the congenic strain genetically close to the strain background of control animals.

5.4 Marker-assisted backcrossing for quality assurance and refinement

The use of DNA markers has allowed for a much more rapid and rigorous process of congenic strain development called *marker-assisted* backcrossing or *speed congenics* [139]. This process relies on using polymorphic genetic markers covering the whole genome to determine the percentage of donor genome present in the animals, then selecting the animals with the lowest percentage of donor DNA for the next

backcross to the recipient strain. This relies on the regions between the polymorphic genetic markers being those of the donor genome: the denser the number of markers the higher the donor genome can be inferred. Common practice is the use of 100-300 markers. This process reduces the number of generations to reach full congenicity (e.g. from N10 to N5), and therefore strain development time, by approximately half. Using marker-assisted backcrosses and the right number of animals we can obtain ~80% recipient background at N2, ~94% at N3, and ~99% at N4 (compared to the classical mean values of 75.0%, 87.5%, and 93.7%). A flowchart depicting a standard speed congenic protocol is shown in **Figure 4**. Ideally, the backcross procedure is started with a donor female and a recipient male. Then, F1 mutant males will carry the correct Y-chromosome and after mating to a recipient female, males of the N2 generation will carry the correct X- and Y-chromosome of the recipient strain (avoiding the use of genetic markers on these chromosomes) [140]. It was predicted by Markel *et al.* that if 20 best breeders (carriers) are used at each generation of the speed congenics protocol >98% recipient genome can be attained at N5 [141] [142]. Also, the 'speed congenic strategy' can be further accelerated by different methods of assisted reproduction [143]. Notably, although a large number of molecular markers are necessary to perform efficient and reliable genotyping during the first backcross generation (N2), this number decreases rapidly because once a marker is typed "homozygous" for the allelic form of the background strain, it is no longer necessary to genotype the offspring of the future generations for this marker because it is permanently fixed.

For projects where the donor strain is unknown or it is a line thought to have more than three different backgrounds, SSLP genotyping, with its great power of discrimination compared with SNP genotyping, should be used. Ideally, markers for speed congenic strain development should include an extra set of polymorphic markers flanking the mutation, transgene, or targeted gene of interest to allow the selection of breeders with the smallest amount of flanking DNA (coming from the ES cell line, the original genome into which a transgene was inserted, or where a spontaneous mutation arose). This helps address a potential issue with a modifier gene that flanks the genetic change [144] [26]. This effort may require breeding many mice to obtain breeders with small introgressed segments.

6. Genetic stability and cryopreservation programmes

For inbred, co-isogenic, and congenic strains, breeding methods and genetic stability programmes help to minimize substrain divergence due to genetic drift, and also to prevent genetic contamination by accidental crosses with other strains. To reduce genetic drift, the number of generations of in-house breeding should be minimized, and the lines submitted to repositories such as, JAX, EMMA, MMRRC, IMSR, or RIKEN, to be archived as frozen embryos and/or sperm. This secures the line and provide a means to replace the breeding stock every 10 generations as recommended by The Jackson Laboratory Genetic Stability Program (GSP) to prevent cumulative genetic

drift [116].

A key to implementing a successful GeMo programme is to invest in education and training of the personnel (See Section 8). Good colony management helps prevent genetic contamination and genetic drift. Caretakers must recognize strain characteristics and animals that deviate from strain norms. Phenotypically indistinguishable inbred strains should not be housed in the same room (or rack), and procedures should be in place to avoid strain mix-ups. To maintain a GA line on a pure inbred background, backcrossing heterozygous carriers with pure inbred mice is recommended. Maintaining and archiving heterozygous mice (e.g., +/-) on inbred backgrounds is ideal even when homozygous or multiple mutants are needed for research, because crossing heterozygotes to produce homozygotes (25% -/-) will also produce proper littermate controls (50% +/-; 25% +/+). Using the correct nomenclature to describe the animal model, and keeping a detailed description of the genetic background is imperative. When planning experiments with multiple lines, it is best to use a common genetic background (e.g., the floxed strain and the Cre-expressing strain on the same background) so that experiments can be replicated.

One recommended scheme for breeding inbred strains is the pyramid-mating scheme. In this system, the foundation colony serves as the genetic and health standard and provides breeders for the top level of the pyramid in every barrier room. This top level, the nucleus colony, is composed of a relatively small number of pedigreed brother-sister mating pairs that produce breeders for the next level of the

pyramid, in addition to replenishing itself. In larger colonies, the next level is called the expansion colony, and it provides breeders to the production colony, which in turn produces the animals for experiments. The unidirectional flow of breed stock in this system helps to ensure that any genetic changes or mutations, which would be more likely to occur in the larger expansion or production colonies than in the smaller nucleus colony, will wash out within a single generation [145].

For outbred stocks, the intent is to minimize inbreeding, maintain heterozygosity and manage genetic drift that would otherwise lead to colony divergence. Ideally, outbred colonies should be maintained with ≥25 breeding pairs, all of which have to contribute to the next generation, in order to avoid an increase of the inbreeding coefficient per generation of more than 1%. Smaller colonies drift fast toward homozygosity because breeders are closely related [146].

If a new GA line is produced on an undefined or incorrect genetic background, it should be backcrossed for ≥ 4-5 generations to the proper genetic background to describe the phenotype [147]. Subsequently, the line should be cryopreserved, the recovery of viable sperm (through IVF) or embryos after thawing should be tested, and the expected genotypes validated. Embryo freezing has traditionally been the method of choice for archiving mouse lines, while sperm freezing is emerging as more convenient alternative, due to the application of innovative Assisted Reproductive Technologies (ARTs) [148] [149] [150]. In the near future, archiving and

exchanging mutant strains between research facilities will require only obtaining, freezing, storing and transporting mouse sperm [151] [152] [153].

Cryopreservation strategies have been adopted for long-term storage of embryos and gametes in several large centralized repositories including the EMMA/INFRAFRONTIER (European Mutant Mouse Archive) [154] [155], the Knock Out Mouse Project (KOMP) Repository [156], The Jackson Laboratory Repository [149], The Center for Animal Resources and Development (CARD) [157] and the Riken Bio Resource Center [158], which can provide cryopreserved material or live mice to laboratories. These repositories facilitate the availability of these strains to the worldwide scientific community and provide a backup for a potential loss of a strain. The International Mouse Strain Resource (IMSR) is a searchable online database of mouse strains, stocks, and mutant ES cell lines available worldwide, including inbred, mutant, and genetically engineered strains (http://www.findmice.org/).

7. Management of rodent facilities

Rodent colony management plans must be carefully considered given the potential for genetic contamination and more classical issues like breeding performance. It is still good practice to separate breeding stocks by coat colour, but comprehensive QA of both the genetic modification and genetic make-up of the strain is imperative. Standard good practice for colony management should consider breeding matters

such as age at mating (e.g., start early at 4-12 weeks of age and replace non-productive breeders), litter size, inter-litter interval, productive breeding life (e.g., retire breeders after 9 months of age, depending on the strain), pre-weaning mortality rates, and phenotypic issues (e.g. sub-viable life expectancy etc.).

7.1 Facility organization

Regarding breeding GA rodents, there will be preferences at individual institutes driven by experimental design, line maintenance costs, and space constraints. Lines may be managed in a variety of genetic combinations, e.g. heterozygous x WT, heterozygous x heterozygous or homozygous intercrosses. The proper choice depends on the required outcome, for example, the need to produce sufficient experimental animals in a set ratio of genotypes, the need to refresh a constituent background, or to reduce the need for genotyping. However, care should be taken to ensure that inadvertent human error or spontaneous mutations have not affected the colony. Both may go unnoticed for several generations becoming obvious only when a deleterious phenotype appears or experimental data varies from what is expected. Key colony management plans must include a genetic integrity component, for example a colony replacement from the pedigree stocks at periodic intervals governed by either a specific number of generations (e.g., 5) or amount of time (2 years). GA colonies should be monitored for breeding, viability, and mutation structure. Any lines that begin to drift from known parameters should be crossed back to the background of choice or returned to the originator to re-establish the

model. Particular consideration should be paid to multi allelic crosses where many generations of crosses and potentially years of research are used to establish these complex models. If possible, key stages should be preserved (archived) as embryos for future recovery of the line and to minimise the effect of any contamination or loss of the mutation involved in the make-up of the model.

7.2. Assisting programmes (ART, cryopreservation, and re-derivation)

Cryopreservation of a model allows a facility to not only offset issues of genetic drift, strain contamination, and pathogen infection, but also to assist in business continuity planning. Single alleles should be preserved as sperm from a carrier male(s). This avoids the unnecessary use of females for lines that may not be recovered and provides for the use of a smaller number of females during *in vitro* fertilization (IVF) protocols. When multi-allelic crosses are used, embryo cryopreservation may be preferable. At the point of preservation, a sample should be taken from the donor animals to confirm the presence of the mutation of interest and whether the documented background contribution matches the expectation upon recovery. Recovery of such models requires the ability to either rederive strains from cryopreserved embryos or through IVF protocols to provide the embryos needed for transfer (it is recommended to seek outside services in case the receiving institution has no embryo technology available). Once recovered, standard animal health checks should be undertaken before releasing the rederived strains into the main breeding or experimental areas of the animal facility/vivarium. Even cryopreservation slows

down the genetic drift of the strain, it is important to carefully plan the number of embryos to be frozen, the number of breeding pairs used to produce the embryos (with very clear genetic information) in order to know the genetic background recovered. It is also advisable to keep frozen tissue or DNA to be able to compare the original genetic background has been recovered.

7.3 Working according to protocol (Standard Operating Procedures)

A structured approach to the management of colonies is essential to ensure accurate data tracking. Structure is provided through Standard Operating Procedures (SOP) for components of the actual breeding, separation, health monitoring, genotyping, phenotyping, cryopreservation, and GeMo of any given line. Data management resources can range from simple paper records when managing one or a few lines, to basic spread sheet and database solutions such as Microsoft Excel and Access, respectively, for colonies in their tens, and other database structures such as FileMaker Pro or complex management systems from commercial software providers when managing 100s to 1000s of mutations and colonies. The latter, when structured properly, allows seamless access to both current and historical data. The choice of records management solution depends on the desired output for a facility. Many solutions contrary to the needs of GA colony management will be driven by the need for budgetary or procedural control and, as such, may omit key information such as breeding performance, pedigree, and experimental data.

When designing or purchasing an Animal Management System (AMS) to manage GA colonies, consider what information will be important to monitor colony performance and track the mutation(s) within the colony. First, consider the unique animal identifier that will appear as an alphanumeric link within a table. This should be converted to a human readable version and convey a sense of the mutation of interest and a link to its parental history. Such identification systems will apply a numerical and alphabetical code to delineate the gene, mating and litter number along with the unique animal code. The minimal information required will be breeding information such as dam and sire identity/genotype, date of set up, birth, weaning and death, litter identity and number born. Genetic information like genotype, background, mutation, gene, pedigree (F and N generation) should provide clear understanding of the gene of interest, the alleles associated to it ideally links to other genetic repositories defining the sequence or origin such as ENSEMBLE and the Mouse Genome Informatics resources (see **Table 2** for links). Welfare and phenotypic information should be understandable to technical, research, and veterinary staff. A shared vocabulary will allow proper recording of data [159]. This may take the form of clinical or pathology ontologies such as the Mouse Pathology Ontology (MPATH) [160]. Other hierarchical lists, such as the Mouse Welfare Terms list, help bridge the gap between the technical and veterinary staff vocabularies, but requires a clear understanding of the application of the observable trait.

Information can be obtained from the very beginning of the data capture during genome engineering, from the targeted sequence to all the stages of development and experimental research. This can be seen in logical steps such as assay development for the genotyping of the mutant animals and into the deeper relationships between individual mutations of a given gene family or metabolic pathway. By structuring the data carefully, phenotypic observations and parameters can be compared and interpreted. As an example, a simple association between the background breeding performance and the allele being studied may reveal reduced pup viability in the presence of the allele. Data from the IMPC shows that around 24% of genes will not produce homozygous offspring (homozygous lethal). Other alleles will confer sub viability from parturition to adult stages and will drive the way in which specific breeding strategies are applied. This is key in ensuring the correct use of such colonies but moreover maintains the concepts and application of the 3R's by reducing and refining animal usage and numbers.

Some of the Software solutions currently (2019) available:

Publicly available:

Jackson Laboratory Colony Management System (JCMS)

(http://colonymanagement.jax.org/)

Sanger MCMS

(https://www.sanger.ac.uk/science/tools/mcms-mouse-colony-management-system)

Commercially available:

eMouseLab (http://e-mouselab.com/Default.aspx)

LabTracks (http://www.locustechnology.com/labtracks.html)

mLIMS (https://bioinforx.com/lims2/product_mlims.php)

PyRAT Animal Facility Software (http://www.mousehouse.io)

SoftMouse Internet Colony Management Software (www.softmouse.net)

AniBio from Noraybio (www.noraybio.com)

Mausoleum (http://www.maus-o-leum.de/)

Tick@lab (https://www.a-tune.com)

Finally, the data should be as comprehensive as possible to allow the producing institute to convey detail regarding the origin development and maintenance of the model. The nomenclature defining the background, gene of interest or mutation, method of mutation and origin via ILAR laboratory codes should be associated with the model. The model should be submitted to a credentialed, sustainable repository, and data made publicly available via collections such as the IMSR, INFRAFRONTIER, the Mutant Mouse Resource & Research Centers (MMRRC) and the IMPC (see Table 2 for links). When depositing a GA model, care should be taken to provide a technical and breeding profile to ensure receiving institutions can manage their own colonies in a manner consistent with that of the original provider.

8. Education

A central element and important focus of the DIRECTIVE 2010/63/EU is the professional qualification and competence of breeders', suppliers', and users' staff. In article 23 (competence of personnel), four functions (points a-d) that require adequate education and training before they can be fulfilled by involved personnel are defined. From scientists responsible for 'designing procedures and projects' (Article 23, paragraph 2 point b) a species-specific, in-depth knowledge of appropriate breeding, genetics, and genetic alteration is expected. A list of elements in Annex V of the Directive refers to Article 23. This FELASA guideline includes 'basic and appropriate species-specific biology in breeding, genetics and genetic alteration' as stated under number 3, which should be considered with the 3R's as outlined under number 10 of Annex V of the DIRECTIVE 2010/63/EU.

Laboratory rodents represent 80-90% of the animals used in research. The most important aspect of designing a research project using laboratory animals is selecting the most appropriate model. For this, scientists must understand the specific genotype and phenotype of the hundreds of standard inbred strains and outbred stocks, plus thousands of mutants that are available. Breeding laboratory rodents is routine in experimental animal facilities, but breeders and suppliers must ensure the genetic quality and integrity of the animal models they provide for example though GeMo and genetic stability programs. However, new GA lines are constantly being generated and established. The biggest challenge in such breeding procedures is

maintaining the mutation on a defined genetic background and recognizing deviant phenotypes, and their severity relative to both the corresponding wild-type and the historic strain-specific norms. The importance and the challenge of this work are often underestimated. With the exception of supervisory and technical staff, animal husbandry is often performed in academic institutions by novices, and job-turnover is high, making it imperative for knowledgeable staff to properly supervise, educate, and train such personnel. Procedures must be well-defined and continuously updated to facilitate training and in accordance with scientific progress.

FELASA accredits education and training courses in laboratory animal science (category A-D). The FELASA accreditation scheme was recently updated according to the four defined functions of persons participating in animal experiments (Directive 2010/63/EU, Article 23, paragraph 2 points a-d). The newly designed courses provide basic knowledge of and initial training in each of the four functions; however, these basics must be extended to lead to full competency. Fundamentals in breeding and genetics as well as phenotype characterization of laboratory animals should be included in the novel Core Modules of functions A-C. In addition, FELASA also provides a list of different types of general and species-specific modules that can be attended to acquire function-specific training, necessary to fulfil tasks within one of the four EU functions (FELASA 2015 Recommendations for the Accreditation of Education and Training Courses in Laboratory Animal Science). Genetics, breeding procedures, quality and integrity testing of laboratory rodents could be part of

Function B: design of procedures and projects. As the list is part of a living document on the FELASA website, a future module with the description ‘Genetic Quality Assurance of Laboratory Rodents’ should also be separately established based on the content of this guideline to create more awareness for the need of proper in-depth training.

9. Sources and further reading

Books available online:

Mouse Genetics: Concepts and Applications. Lee M. Silver, Oxford University Press, 1995. http://www.informatics.jax.org/silver/

Origins of Inbred Mice. Herbert C. Morse III, Academic Press, 1978. http://www.informatics.jax.org/morsebook/

The Coat Colors of Mice. Willys K. Silvers, Springer Verlag, 1979. http://www.informatics.jax.org/wksilvers/

(in Spanish). Manual de Genética de Roedores de Laboratorio. Principios básicos y aplicaciones. Fernando Benavides, Jean-Louis Guénet. Laboratory Animals Ltd, 2003. https://secal.es/publicaciones/libros/

Reference Books:

ICLAS Manual for Genetic Monitoring of Inbred Mice. Nomura T, Univ of Tokyo Press, 1984.

Genetic Monitoring of Inbred Strains of Rats. Hans J Hedrich, Gustav Fischer Verlag, 1990.

Genetics of the Mouse. Jean Louis Guénet, Fernando Benavides, Jean-Jacques Panthier, Xavier Montagutelli, Springer, 2015.

The Laboratory Mouse (2nd edition). Hans J Hedrich. Academic Press, 2012.

The Laboratory Rat. George Krinke, Academic Press, 2000.

The Laboratory Rat (2nd edition). Mark Suckow, Steven Weisbroth, Craig Franklin, Academic Press, 2005.

Rat Genomics. Methods and Protocols. Ignacio Anegón, Springer, 2010.

10. References

1. Silver L. *Mouse Genetics. Concepts and Applications*. Oxford University Press, 1995.
2. Simecek P, Forejt J, Williams RW, et al. High-Resolution Maps of Mouse Reference Populations. *G3 (Bethesda)*. 2017; 7: 3427-34.
3. Guenet JL, Benavides, F., Panthier, J., Montagutelli, X. *Genetics of the Mouse*. Springer, 2015.
4. Paigen K and Eppig JT. A mouse phenome project. *Mamm Genome*. 2000; 11: 715-7.
5. Eppig JT. Mouse Genome Informatics (MGI) Resource: Genetic, Genomic, and Biological Knowledgebase for the Laboratory Mouse. *ILAR J*. 2017; 58: 17-41.
6. Waterston RH, Lindblad-Toh K, Birney E, et al. Initial sequencing and comparative analysis of the mouse genome. *Nature*. 2002; 420: 520-62.
7. Gibbs RA, Weinstock GM, Metzker ML, et al. Genome sequence of the Brown Norway rat yields insights into mammalian evolution. *Nature*. 2004; 428: 493-521.
8. Adams DJ, Doran AG, Lilue J and Keane TM. *The Mouse Genomes Project: a repository of inbred laboratory mouse strain genomes*. 2015, p.403-12.
9. Frazer KA, Eskin E, Kang HM, et al. A sequence-based variation map of 8.27 million SNPs in inbred mouse strains. *Nature*. 2007; 448: 1050-3.
10. Yang H, Wang JR, Didion JP, et al. Subspecific origin and haplotype diversity in the laboratory mouse. *Nat Genet*. 2011; 43: 648-55.
11. Bello SM, Smith CL and Eppig JT. Allele, phenotype and disease data at Mouse Genome Informatics: improving access and analysis. *Mamm Genome*. 2015; 26: 285-94.
12. Bucan M, Eppig JT and Brown S. Mouse genomics programs and resources. *Mamm Genome*. 2012; 23: 479-89.
13. Eppig JT, Blake JA, Bult CJ, Kadin JA, Richardson JE and Mouse Genome Database G. The Mouse Genome Database (MGD): comprehensive resource for genetics and genomics of the laboratory mouse. *Nucleic Acids Res*. 2012; 40: D881-6.
14. Takada T, Yoshiki A, Obata Y, Yamazaki Y and Shiroishi T. NIG_MoG: a mouse genome navigator for exploring intersubspecific genetic polymorphisms. *Mamm Genome*. 2015; 26: 331-7.
15. Zhu Y, Richardson JE, Hale P, et al. A unified gene catalog for the laboratory mouse reference genome. *Mamm Genome*. 2015; 26: 295-304.
16. Brown SDM, Holmes CC, Mallon AM, Meehan TF, Smedley D and Wells S. High-throughput mouse phenomics for characterizing mammalian gene function. *Nat Rev Genet*. 2018; 19: 357-70.
17. Hartl DL. *Genetic Management of outbred laboratory rodent populations.*: Charles River Genetic Literature, 2001.
18. Poiley SM. A systematic method of breeder rotation for non-inbred laboratory animals colonies. *Proc Anim Care Panel*. 1960; 10: 159.
19. Falconer DS. Genetic aspects of breeding methods. In: Lane-Petter W, (ed.). *UFAW Handbook on the Care and Management of Laboratory Animals*. 3rd ed. Edinburgh, UK: Livingstone, Edinburgh, 1967, p. 72-96.

20. Eggenberger E. Model populations for assessment of rotation systems in experimental animal breeding. *Z Versuchstierkd* 1973; 15: 297-331.
21. Chia R, Achilli F, Festing MF and Fisher EM. The origins and uses of mouse outbred stocks. *Nat Genet*. 2005; 37: 1181-6.
22. Festing MF. Inbred strains should replace outbred stocks in toxicology, safety testing, and drug development. *Toxicol Pathol*. 2010; 38: 681-90.
23. Yalcin B, Nicod J, Bhomra A, et al. Commercially available outbred mice for genome-wide association studies. *PLoS Genet*. 2010; 6: e1001085.
24. Churchill GA, Gatti DM, Munger SC and Svenson KL. The Diversity Outbred mouse population. *Mamm Genome*. 2012; 23: 713-8.
25. Tuttle AH, Philip VM, Chesler EJ and Mogil JS. Comparing phenotypic variation between inbred and outbred mice. *Nat Methods*. 2018; 15: 994-6.
26. Chen S, Kadomatsu K, Kondo M, et al. Effects of flanking genes on the phenotypes of mice deficient in basigin/CD147. *Biochem Biophys Res Commun*. 2004; 324: 147-53.
27. Schuster-Gossler K, Lee AW, Lerner CP, et al. Use of coisogenic host blastocysts for efficient establishment of germline chimeras with C57BL/6J ES cell lines. *Biotechniques*. 2001; 31: 1022-4, 6.
28. Nadeau JH, Singer JB, Matin A and Lander ES. Analysing complex genetic traits with chromosome substitution strains. *Nat Genet*. 2000; 24: 221-5.
29. Bailey DW. Recombinant-inbred strains. An aid to finding identity, linkage, and function of histocompatibility and other genes. *Transplantation*. 1971; 11: 325-7.
30. Churchill GA, Airey DC, Allayee H, et al. The Collaborative Cross, a community resource for the genetic analysis of complex traits. *Nat Genet*. 2004; 36: 1133-7.
31. Threadgill DW and Churchill GA. Ten years of the collaborative cross. *G3 (Bethesda)*. 2012; 2: 153-6.
32. Hogan B, Beddington, R., Costantini, F., Lacy, E. . *Manipulating the mouse embryo: a laboratory manual, 2nd edn.*: Cold Spring Harbor Laboratory Press, Cold Spring Harbor, 1994.
33. Jackson IJ, Abbott, C.M. . *Mouse genetics and transgenesis: a practical approach*. Oxford University Press, Oxford, 2000.
34. Nagy A, Gertsenstein, M., Vintersten, K., Behringer, R. . *Manipulating the mouse embryo, a laboratory manual, 3rd edn.*: Cold Spring Harbor Press, New York, 2003.
35. Koentgen F, Suess G and Naf D. Engineering the mouse genome to model human disease for drug discovery. *Methods Mol Biol*. 2010; 602: 55-77.
36. Brinster RL, Chen HY, Trumbauer M, Senear AW, Warren R and Palmiter RD. Somatic expression of herpes thymidine kinase in mice following injection of a fusion gene into eggs. *Cell*. 1981; 27: 223-31.
37. Costantini F and Lacy E. Introduction of a rabbit beta-globin gene into the mouse germ line. *Nature*. 1981; 294: 92-4.
38. Gordon JW and Ruddle FH. Integration and stable germ line transmission of genes injected into mouse pronuclei. *Science*. 1981; 214: 1244-6.

39. Ballester M, Castello A, Ibanez E, Sanchez A and Folch JM. Real-time quantitative PCR-based system for determining transgene copy number in transgenic animals. *Biotechniques*. 2004; 37: 610-3.
40. Taketo M, Schroeder AC, Mobraaten LE, et al. FVB/N: an inbred mouse strain preferable for transgenic analyses. *Proc Natl Acad Sci U S A*. 1991; 88: 2065-9.
41. Gossen M and Bujard H. Tight control of gene expression in mammalian cells by tetracycline-responsive promoters. *Proc Natl Acad Sci U S A*. 1992; 89: 5547-51.
42. Baron U and Bujard H. Tet repressor-based system for regulated gene expression in eukaryotic cells: principles and advances. *Methods Enzymol*. 2000; 327: 401-21.
43. Lois C, Hong EJ, Pease S, Brown EJ and Baltimore D. Germline transmission and tissue-specific expression of transgenes delivered by lentiviral vectors. *Science*. 2002; 295: 868-72.
44. Ivics Z, Mates L, Yau TY, et al. Germline transgenesis in rodents by pronuclear microinjection of Sleeping Beauty transposons. *Nat Protoc*. 2014; 9: 773-93.
45. Perry AC, Wakayama T, Kishikawa H, et al. Mammalian transgenesis by intracytoplasmic sperm injection. *Science*. 1999; 284: 1180-3.
46. Fernandez-Gonzalez R, Moreira PN, Perez-Crespo M, et al. Long-term effects of mouse intracytoplasmic sperm injection with DNA-fragmented sperm on health and behavior of adult offspring. *Biol Reprod*. 2008; 78: 761-72.
47. Li P, Tong C, Mehrian-Shai R, et al. Germline competent embryonic stem cells derived from rat blastocysts. *Cell*. 2008; 135: 1299-310.
48. Sauer B and Henderson N. Site-specific DNA recombination in mammalian cells by the Cre recombinase of bacteriophage P1. *Proc Natl Acad Sci U S A*. 1988; 85: 5166-70.
49. Buchholz F, Angrand PO and Stewart AF. Improved properties of FLP recombinase evolved by cycling mutagenesis. *Nat Biotechnol*. 1998; 16: 657-62.
50. Sauer B and McDermott J. DNA recombination with a heterospecific Cre homolog identified from comparison of the pac-c1 regions of P1-related phages. *Nucleic Acids Res*. 2004; 32: 6086-95.
51. Feil S, Valtcheva N and Feil R. Inducible Cre mice. *Methods Mol Biol*. 2009; 530: 343-63.
52. Danielian PS, Muccino D, Rowitch DH, Michael SK and McMahon AP. Modification of gene activity in mouse embryos in utero by a tamoxifen-inducible form of Cre recombinase. *Curr Biol*. 1998; 8: 1323-6.
53. Wagner KU, McAllister K, Ward T, Davis B, Wiseman R and Hennighausen L. Spatial and temporal expression of the Cre gene under the control of the MMTV-LTR in different lines of transgenic mice. *Transgenic Res*. 2001; 10: 545-53.
54. Kaneko T and Mashimo T. Creating knockout and knockin rodents using engineered endonucleases via direct embryo injection. *Methods Mol Biol*. 2015; 1239: 307-15.
55. Geurts AM, Cost GJ, Freyvert Y, et al. Knockout rats via embryo microinjection of zinc-finger nucleases. *Science*. 2009; 325: 433.

56. Carbery ID, Ji D, Harrington A, et al. Targeted genome modification in mice using zinc-finger nucleases. *Genetics*. 2010; 186: 451-9.
57. Cui X, Ji D, Fisher DA, Wu Y, Briner DM and Weinstein EJ. Targeted integration in rat and mouse embryos with zinc-finger nucleases. *Nat Biotechnol*. 2011; 29: 64-7.
58. Mashimo T. Gene targeting technologies in rats: zinc finger nucleases, transcription activator-like effector nucleases, and clustered regularly interspaced short palindromic repeats. *Dev Growth Differ*. 2014; 56: 46-52.
59. Joung JK and Sander JD. TALENs: a widely applicable technology for targeted genome editing. *Nat Rev Mol Cell Biol*. 2013; 14: 49-55.
60. Sung YH, Baek IJ, Kim DH, et al. Knockout mice created by TALEN-mediated gene targeting. *Nat Biotechnol*. 2013; 31: 23-4.
61. Jinek M, Chylinski K, Fonfara I, Hauer M, Doudna JA and Charpentier E. A programmable dual-RNA-guided DNA endonuclease in adaptive bacterial immunity. *Science*. 2012; 337: 816-21.
62. Pennisi E. The CRISPR craze. *Science*. 2013; 341: 833-6.
63. Yang H, Wang H, Shivalila CS, Cheng AW, Shi L and Jaenisch R. One-step generation of mice carrying reporter and conditional alleles by CRISPR/Cas-mediated genome engineering. *Cell*. 2013; 154: 1370-9.
64. Wang H, Yang H, Shivalila CS, et al. One-step generation of mice carrying mutations in multiple genes by CRISPR/Cas-mediated genome engineering. *Cell*. 2013; 153: 910-8.
65. Horii T, Arai Y, Yamazaki M, et al. Validation of microinjection methods for generating knockout mice by CRISPR/Cas-mediated genome engineering. *Sci Rep*. 2014; 4: 4513.
66. Guan Y, Shao Y, Li D and Liu M. Generation of site-specific mutations in the rat genome via CRISPR/Cas9. *Methods Enzymol*. 2014; 546: 297-317.
67. Shao Y, Guan Y, Wang L, et al. CRISPR/Cas-mediated genome editing in the rat via direct injection of one-cell embryos. *Nat Protoc*. 2014; 9: 2493-512.
68. Yoshimi K, Kaneko T, Voigt B and Mashimo T. Allele-specific genome editing and correction of disease-associated phenotypes in rats using the CRISPR-Cas platform. *Nat Commun*. 2014; 5: 4240.
69. Hsu PD, Lander ES and Zhang F. Development and applications of CRISPR-Cas9 for genome engineering. *Cell*. 2014; 157: 1262-78.
70. Zhang F, Wen Y and Guo X. CRISPR/Cas9 for genome editing: progress, implications and challenges. *Hum Mol Genet*. 2014; 23: R40-6.
71. Seruggia D, Fernandez A, Cantero M, Pelczar P and Montoliu L. Functional validation of mouse tyrosinase non-coding regulatory DNA elements by CRISPR-Cas9-mediated mutagenesis. *Nucleic Acids Res*. 2015; 43: 4855-67.
72. Fernandez A, Josa S and Montoliu L. A history of genome editing in mammals. *Mamm Genome*. 2017; 28: 237-46.
73. Miyasaka Y, Uno Y, Yoshimi K, et al. CLICK: one-step generation of conditional knockout mice. *BMC Genomics*. 2018; 19: 318.

74. Chen S, Lee B, Lee AY, Modzelewski AJ and He L. Highly Efficient Mouse Genome Editing by CRISPR Ribonucleoprotein Electroporation of Zygotes. *J Biol Chem*. 2016; 291: 14457-67.
75. Gu B, Posfai E and Rossant J. Efficient generation of targeted large insertions by microinjection into two-cell-stage mouse embryos. *Nat Biotechnol*. 2018; 36: 632-7.
76. Cameron P, Fuller CK, Donohoue PD, et al. Mapping the genomic landscape of CRISPR-Cas9 cleavage. *Nat Methods*. 2017; 14: 600-6.
77. Kosicki M, Tomberg K and Bradley A. Repair of double-strand breaks induced by CRISPR-Cas9 leads to large deletions and complex rearrangements. *Nat Biotechnol*. 2018; 36: 765-71.
78. Rezza A, Jacquet C, Le Pillouer A, et al. Unexpected genomic rearrangements at targeted loci associated with CRISPR/Cas9-mediated knock-in. *Sci Rep*. 2019; 9: 3486.
79. Nolan PM, Peters J, Strivens M, et al. A systematic, genome-wide, phenotype-driven mutagenesis programme for gene function studies in the mouse. *Nat Genet*. 2000; 25: 440-3.
80. Bonaparte D, Cinelli P, Douni E, et al. FELASA guidelines for the refinement of methods for genotyping genetically-modified rodents: a report of the Federation of European Laboratory Animal Science Associations Working Group. *Lab Anim*. 2013; 47: 134-45.
81. Rulicke T, Montagutelli X, Pintado B, Thon R and Hedrich HJ. FELASA guidelines for the production and nomenclature of transgenic rodents. *Lab Anim*. 2007; 41: 301-11.
82. Ivics Z, Izsvak Z, Chapman KM and Hamra FK. Sleeping Beauty transposon mutagenesis of the rat genome in spermatogonial stem cells. *Methods*. 2011; 53: 356-65.
83. Yen ST, Zhang M, Deng JM, et al. Somatic mosaicism and allele complexity induced by CRISPR/Cas9 RNA injections in mouse zygotes. *Dev Biol*. 2014; 393: 3-9.
84. Coleman DL and Hummel KP. The influence of genetic background on the expression of the obese (Ob) gene in the mouse. *Diabetologia*. 1973; 9: 287-93.
85. Kurtz TW, Montano M, Chan L and Kabra P. Molecular evidence of genetic heterogeneity in Wistar-Kyoto rats: implications for research with the spontaneously hypertensive rat. *Hypertension*. 1989; 13: 188-92.
86. St Lezin EM, Pravenec M, Wong A, et al. Genetic contamination of Dahl SS/Jr rats. Impact on studies of salt-sensitive hypertension. *Hypertension*. 1994; 23: 786-90.
87. Fairfield H, Srivastava A, Ananda G, et al. Exome sequencing reveals pathogenic mutations in 91 strains of mice with Mendelian disorders. *Genome Res*. 2015; 25: 948-57.
88. Peters H, Reifenberg K, Wedekind, D. Substrains of Inbred Strains. *GV-SOLAS*. 2013; Specialist Information.
89. Mekada K, Abe K, Murakami A, et al. Genetic differences among C57BL/6 substrains. *Exp Anim*. 2009; 58: 141-9.
90. Mekada K, Hirose M, Murakami A and Yoshiki A. Development of SNP markers for C57BL/6N-derived mouse inbred strains. *Exp Anim*. 2015; 64: 91-100.

91. Simpson EM, Linder CC, Sargent EE, Davisson MT, Mobraaten LE and Sharp JJ. Genetic variation among 129 substrains and its importance for targeted mutagenesis in mice. *Nat Genet*. 1997; 16: 19-27.
92. Hermsen R, de Ligt J, Spee W, et al. Genomic landscape of rat strain and substrain variation. *BMC Genomics*. 2015; 16: 357.
93. Kenneth NS, Younger JM, Hughes ED, et al. An inactivating caspase 11 passenger mutation originating from the 129 murine strain in mice targeted for c-IAP1. *Biochem J*. 2012; 443: 355-9.
94. Stevens JC, Banks GT, Festing MF and Fisher EM. Quiet mutations in inbred strains of mice. *Trends Mol Med*. 2007; 13: 512-9.
95. Specht CG and Schoepfer R. Deletion of the alpha-synuclein locus in a subpopulation of C57BL/6J inbred mice. *BMC Neurosci*. 2001; 2: 11.
96. Specht CG and Schoepfer R. Deletion of multimerin-1 in alpha-synuclein-deficient mice. *Genomics*. 2004; 83: 1176-8.
97. Chen PE, Specht CG, Morris RG and Schoepfer R. Spatial learning is unimpaired in mice containing a deletion of the alpha-synuclein locus. *Eur J Neurosci*. 2002; 16: 154-8.
98. Freeman HC, Hugill A, Dear NT, Ashcroft FM and Cox RD. Deletion of nicotinamide nucleotide transhydrogenase: a new quantitive trait locus accounting for glucose intolerance in C57BL/6J mice. *Diabetes*. 2006; 55: 2153-6.
99. Mattapallil MJ, Wawrousek EF, Chan CC, et al. The Rd8 mutation of the Crb1 gene is present in vendor lines of C57BL/6N mice and embryonic stem cells, and confounds ocular induced mutant phenotypes. *Invest Ophthalmol Vis Sci*. 2012; 53: 2921-7.
100. Kumar V, Kim K, Joseph C, et al. C57BL/6N mutation in cytoplasmic FMRP interacting protein 2 regulates cocaine response. *Science*. 2013; 342: 1508-12.
101. Simon MM, Greenaway S, White JK, et al. A comparative phenotypic and genomic analysis of C57BL/6J and C57BL/6N mouse strains. *Genome Biol*. 2013; 14: R82.
102. Poltorak A, He X, Smirnova I, et al. Defective LPS signaling in C3H/HeJ and C57BL/10ScCr mice: mutations in Tlr4 gene. *Science*. 1998; 282: 2085-8.
103. Vanden Berghe T, Hulpiau P, Martens L, et al. Passenger Mutations Confound Interpretation of All Genetically Modified Congenic Mice. *Immunity*. 2015; 43: 200-9.
104. Guenet JL and Benavides FJ. Mouse Strains and Genetic Nomenclature. *Curr Protoc Mouse Biol*. 2011; 1: 213-38.
105. Benavides F, Cazalla D, Pereira C, et al. Evidence of genetic heterogeneity in a BALB/c mouse colony as determined by DNA fingerprinting. *Lab Anim*. 1998; 32: 80-5.
106. Otsen M, Den Bieman M, Winer ES, et al. Use of simple sequence length polymorphisms for genetic characterization of rat inbred strains. *Mamm Genome*. 1995; 6: 595-601.
107. Benavides F, Glasscock E, Coghlan LG, Stern MC, Weiss DA and Conti CJ. PCR-based microsatellite analysis for differentiation and genetic monitoring of nine inbred SENCAR mouse strains. *Lab Anim*. 2001; 35: 157-62.

108. Mashimo T, Voigt B, Tsurumi T, et al. A set of highly informative rat simple sequence length polymorphism (SSLP) markers and genetically defined rat strains. *BMC Genet*. 2006; 7: 19.
109. Petkov PM, Ding Y, Cassell MA, et al. An efficient SNP system for mouse genome scanning and elucidating strain relationships. *Genome Res*. 2004; 14: 1806-11.
110. Myakishev MV, Khripin Y, Hu S and Hamer DH. High-throughput SNP genotyping by allele-specific PCR with universal energy-transfer-labeled primers. *Genome Res*. 2001; 11: 163-9.
111. Zimdahl H, Nyakatura G, Brandt P, et al. A SNP map of the rat genome generated from cDNA sequences. *Science*. 2004; 303: 807.
112. Smits BM, Guryev V, Zeegers D, Wedekind D, Hedrich HJ and Cuppen E. Efficient single nucleotide polymorphism discovery in laboratory rat strains using wild rat-derived SNP candidates. *BMC Genomics*. 2005; 6: 170.
113. Consortium S, Saar K, Beck A, et al. SNP and haplotype mapping for genetic analysis in the rat. *Nat Genet*. 2008; 40: 560-6.
114. Nijman IJ, Kuipers S, Verheul M, Guryev V and Cuppen E. A genome-wide SNP panel for mapping and association studies in the rat. *BMC Genomics*. 2008; 9: 95.
115. Moran JL, Bolton AD, Tran PV, et al. Utilization of a whole genome SNP panel for efficient genetic mapping in the mouse. *Genome Res*. 2006; 16: 436-40.
116. Taft RA, Davisson M and Wiles MV. Know thy mouse. *Trends Genet*. 2006; 22: 649-53.
117. Reardon S. Lab mice's ancestral 'Eve' gets her genome sequenced. *Nature*. 2017; 551: 281.
118. Sarsani VK, Raghupathy N, Fiddes IT, et al. The Genome of C57BL/6J "Eve", the Mother of the Laboratory Mouse Genome Reference Strain. *G3 (Bethesda)*. 2019; 9: 1795-805.
119. Fahey JR, Katoh H, Malcolm R and Perez AV. The case for genetic monitoring of mice and rats used in biomedical research. *Mamm Genome*. 2013; 24: 89-94.
120. Silvers WK. *The Coat Colors of Mice. A Model for Mammalian Gene Action and Interaction*. Springer, 1979.
121. Lamoreux ML, Delams, V., Larue, L., Bennet, D.C. *The colors of mice. A model genetic network.* . Wiley-Blackwell, 2010.
122. Lambert R. *Breeding strategies for maintaining colonies of laboratory mice: a Jackson Laboratory resource manual.* Bar Harbor, Maine, USA: The Jackson Laboratory 2009.
123. Leiter EH, Prochazka M and Coleman DL. The non-obese diabetic (NOD) mouse. *Am J Pathol*. 1987; 128: 380-3.
124. Gaudet M, Fara AG, Beritognolo I and Sabatti M. Allele-specific PCR in SNP genotyping. *Methods Mol Biol*. 2009; 578: 415-24.
125. Yang H, Ding Y, Hutchins LN, et al. A customized and versatile high-density genotyping array for the mouse. *Nat Methods*. 2009; 6: 663-6.
126. Morgan AP, Fu CP, Kao CY, et al. The Mouse Universal Genotyping Array: From Substrains to Subspecies. *G3 (Bethesda)*. 2015; 6: 263-79.

127. Didion JP, Buus RJ, Naghashfar Z, Threadgill DW, Morse HC, 3rd and de Villena FP. SNP array profiling of mouse cell lines identifies their strains of origin and reveals cross-contamination and widespread aneuploidy. *BMC Genomics*. 2014; 15: 847.
128. Zurita E, Chagoyen M, Cantero M, et al. Genetic polymorphisms among C57BL/6 mouse inbred strains. *Transgenic Res*. 2011; 20: 481-9.
129. Linder CC. The influence of genetic background on spontaneous and genetically engineered mouse models of complex diseases. *Lab Anim (NY)*. 2001; 30: 34-9.
130. Doetschman T. Influence of genetic background on genetically engineered mouse phenotypes. *Methods Mol Biol*. 2009; 530: 423-33.
131. Hummel KP, Coleman DL and Lane PW. The influence of genetic background on expression of mutations at the diabetes locus in the mouse. I. C57BL-KsJ and C57BL-6J strains. *Biochem Genet*. 1972; 7: 1-13.
132. Threadgill DW, Dlugosz AA, Hansen LA, et al. Targeted disruption of mouse EGF receptor: effect of genetic background on mutant phenotype. *Science*. 1995; 269: 230-4.
133. Kuperwasser C, Hurlbut GD, Kittrell FS, et al. Development of spontaneous mammary tumors in BALB/c p53 heterozygous mice. A model for Li-Fraumeni syndrome. *Am J Pathol*. 2000; 157: 2151-9.
134. Freeman D, Lesche R, Kertesz N, et al. Genetic background controls tumor development in PTEN-deficient mice. *Cancer Res*. 2006; 66: 6492-6.
135. Bourdi M, Davies JS and Pohl LR. Mispairing C57BL/6 substrains of genetically engineered mice and wild-type controls can lead to confounding results as it did in studies of JNK2 in acetaminophen and concanavalin A liver injury. *Chem Res Toxicol*. 2011; 24: 794-6.
136. Asamoto M, Hokaiwado N, Cho YM and Shirai T. Effects of genetic background on prostate and taste bud carcinogenesis due to SV40 T antigen expression under probasin gene promoter control. *Carcinogenesis*. 2002; 23: 463-7.
137. Fernandez A, Josa S and Montoliu L. A history of genome editing in mammals. *Mamm Genome*. 2017.
138. Wells DJ, Playle LC, Enser WE, et al. Assessing the welfare of genetically altered mice. *Lab Anim*. 2006; 40: 111-4.
139. Wakeland E, Morel L, Achey K, Yui M and Longmate J. Speed congenics: a classic technique in the fast lane (relatively speaking). *Immunol Today*. 1997; 18: 472-7.
140. Dobrowolski P, Fischer M and Naumann R. Novel insights into the genetic background of genetically modified mice. *Transgenic Res*. 2018; 27: 265-75.
141. Markel P, Shu P, Ebeling C, et al. Theoretical and empirical issues for marker-assisted breeding of congenic mouse strains. *Nat Genet*. 1997; 17: 280-4.
142. Gurumurthy CB, Joshi PS, Kurz SG, et al. Validation of simple sequence length polymorphism regions of commonly used mouse strains for marker assisted speed congenics screening. *Int J Genomics*. 2015; 2015: 735845.
143. Ogonuki N, Inoue K, Hirose M, et al. A high-speed congenic strategy using first-wave male germ cells. *PLoS One*. 2009; 4: e4943.

144. Wolfer DP, Crusio WE and Lipp HP. Knockout mice: simple solutions to the problems of genetic background and flanking genes. *Trends Neurosci*. 2002; 25: 336-40.
145. Hedrich HJ. *The Laboratory Mouse*. Second ed.: Elsevier, 2012, p.845.
146. Berry MM, Linder, C.C. Breeding Systems: Considerations, Genetic Fundamentals, Genetic Background and Strain Types. In: Fox JG BS, Davisson MT, Newcomer CE, Quimby FE, and Smith AL, (ed.). *The Mouse in Biomedical Research Volume 1 History, Wild Mice and Genetics* Academic Press, 2007, p. 53-78.
147. Banbury. Mutant mice and neuroscience: Recommendations concerning genetic background: Banbury Conference on genetic background in mice. *Neuron*. 1997; 19: 755-9.
148. Sztein JM, Farley JS and Mobraaten LE. In vitro fertilization with cryopreserved inbred mouse sperm. *Biol Reprod*. 2000; 63: 1774-80.
149. Ostermeier GC, Wiles MV, Farley JS and Taft RA. Conserving, distributing and managing genetically modified mouse lines by sperm cryopreservation. *PLoS One*. 2008; 3: e2792.
150. Takeo T and Nakagata N. Reduced glutathione enhances fertility of frozen/thawed C57BL/6 mouse sperm after exposure to methyl-beta-cyclodextrin. *Biol Reprod*. 2011; 85: 1066-72.
151. Raspa M, Fray M, Paoletti R, et al. Long term maintenance of frozen mouse spermatozoa at -80 degrees C. *Theriogenology*. 2018; 107: 41-9.
152. Moreno-Del Val G and Munoz-Robledano P. Revitalizing genetically-modified mouse strains using frozen-thawed sperm after up to 192 h of refrigerated epididymis transportation. *Lab Anim*. 2017; 51: 526-9.
153. Raspa M, Fray M, Paoletti R, et al. A new, simple and efficient liquid nitrogen free method to cryopreserve mouse spermatozoa at -80 degrees C. *Theriogenology*. 2018; 119: 52-9.
154. Consortium I. INFRAFRONTIER--providing mutant mouse resources as research tools for the international scientific community. *Nucleic Acids Res*. 2015; 43: D1171-5.
155. Wilkinson P, Sengerova J, Matteoni R, et al. EMMA--mouse mutant resources for the international scientific community. *Nucleic Acids Res*. 2010; 38: D570-6.
156. Lloyd KC. A knockout mouse resource for the biomedical research community. *Ann N Y Acad Sci*. 2011; 1245: 24-6.
157. Nakagata N and Yamamura K. Current activities of CARD as an international core center for mouse resources. *Exp Anim*. 2009; 58: 343-50.
158. Yoshiki A, Ike F, Mekada K, et al. The mouse resources at the RIKEN BioResource center. *Exp Anim*. 2009; 58: 85-96.
159. Fentener van Vlissingen JM, Borrens M, Girod A, Lelovas P, Morrison F and Torres YS. The reporting of clinical signs in laboratory animals: FELASA Working Group Report. *Lab Anim*. 2015; 49: 267-83.
160. Schofield PN, Sundberg JP, Sundberg BA, McKerlie C and Gkoutos GV. The mouse pathology ontology, MPATH; structure and applications. *J Biomed Semantics*. 2013; 4: 18.

Figure 1

Backcrosses to generate congenic strains

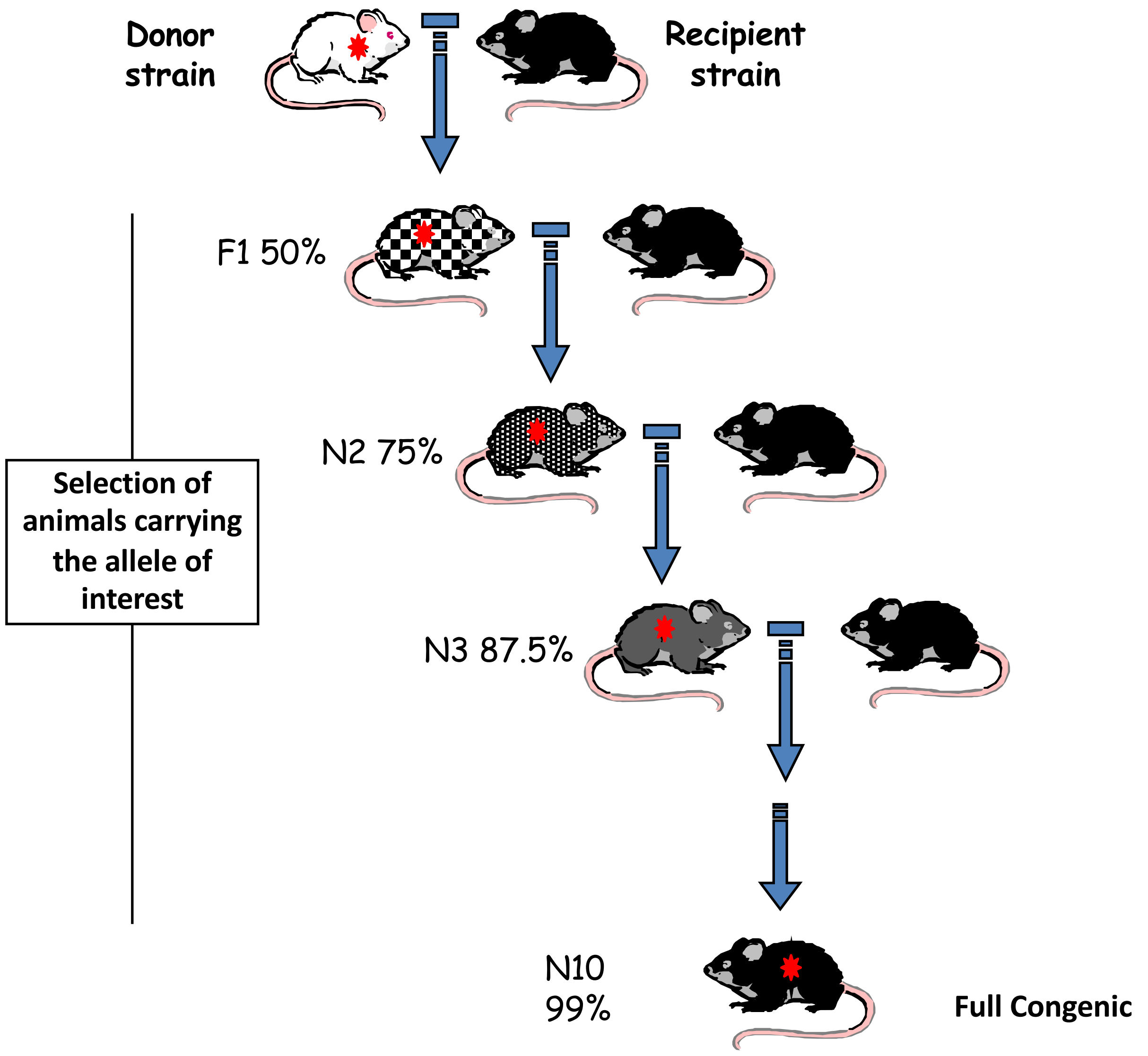

This scheme represents the successive steps in the establishment of a congenic strain. The initial step is a cross between the donor strain (albino in the example) carrying the gene of interest (e.g., a targeted gene or a transgene) and a recipient or background strain (black in the example). At each generation, a breeder carrying the gene of interest (*) is backcrossed to a partner of the recipient strain (genetically linked genes are transferred with it and the size of the introgressed fragment can be many Mb, and include many genes). The degree of gray color indicates that, after each backcross generation, the offspring have an increased amount of the background genome (average percentage is indicated in each N generation). When the targeted gene has no easily recognizable phenotype, molecular genotyping is necessary to select the carrier (heterozygous) mice.

Figure 2

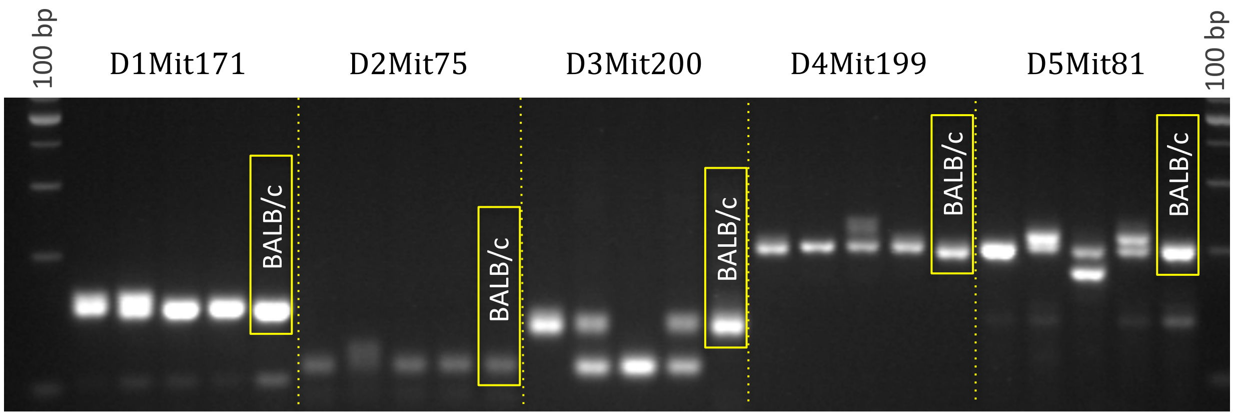

Example of genetic contamination detected by SSLP PCR. The picture shows a 4% agarose gel with the characteristic bands obtained after PCR amplification using genomic DNA from four mice supposedly belonging to the BALB/c strain (first four lanes), plus a standard DNA control for BALB/c (last lane).In this example, only five SSLP loci are shown, located in chromosomes 1 to 5. Note the presence of heterozygosity (two bands) and homozygosity for bands that do not match the standard for BALB/c. This is a clear case of loss of authenticity due to genetic contamination. The PCR products are compared with a 100 bp DNA ladder.

Figure 3

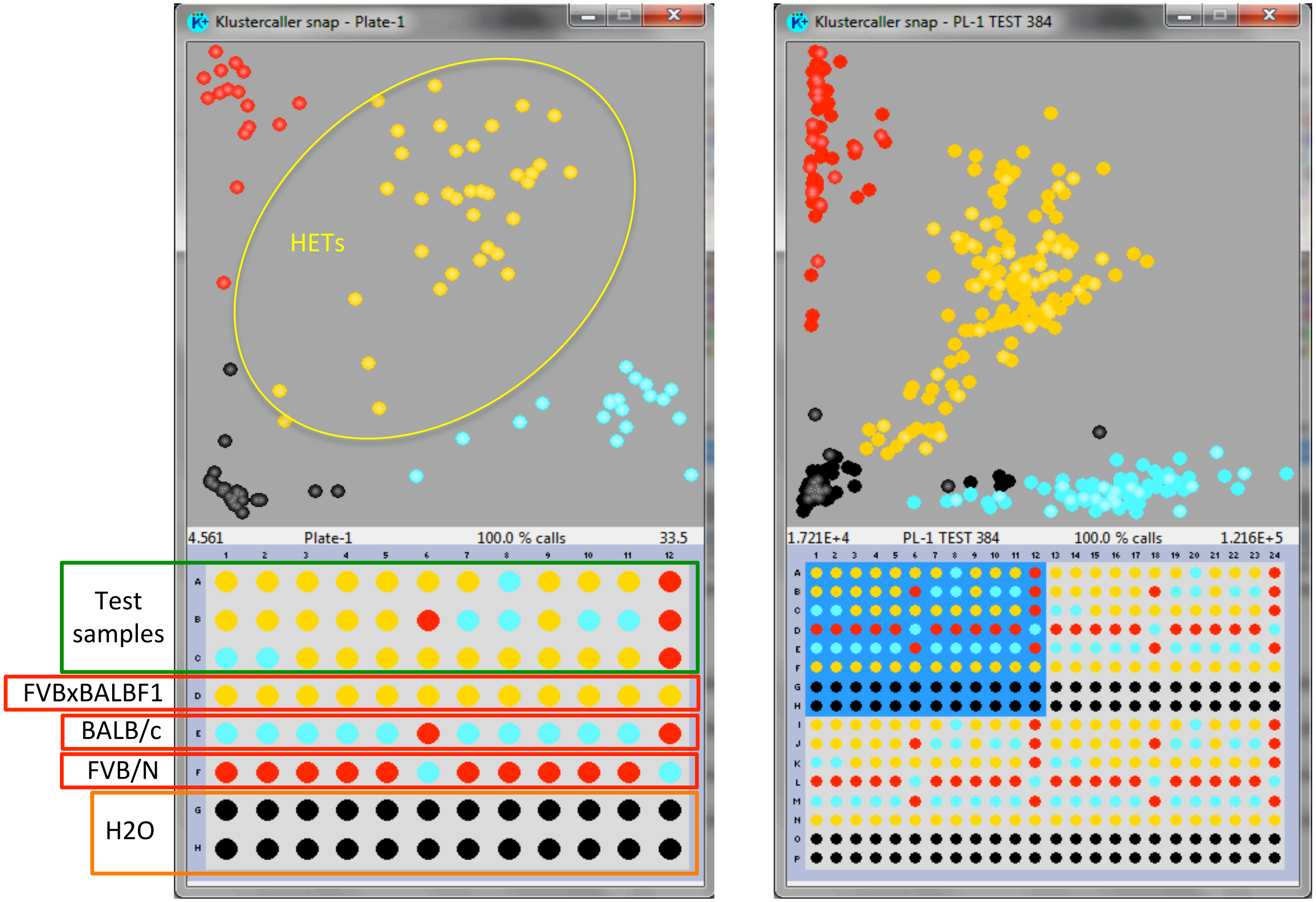

SNP analysis. The figures show results obtained with the KlusterCaller™ software (LGC) after PCR amplification using KASP assays (LGC). When dual emission genotyping data from a fluorescent reader is imported into the KlusterCaller, proprietary algorithms built into the software automatically discern if the assay results are homozygous for allele 1 (red), homozygous for allele 2 (light blue) or heterozygous (yellow). In this example, 12 SNPs were analyzed (columns) in a 96-well plate (left), and the same layout (four replicates) in a 384-well plate (right). Both plates were loaded with an Eppendorf pipetting station. In the 96-well plate, rows A to C are test samples; row D is an (FVBxBALB/c)F1 control; row E is a BALB/c control, and row F is an FVB/N control. Yellow dots represent heterozygous SNP genotypes. Red or light blue dots represent homozygous genotypes (but not necessary a particular strain). Black dots are negative (water) controls. For example, the DNA sample in row A is heterozygous for SNPs 1-7 and 9-11, and homozygous for SNPs 8 and 12 (both BALB/c-like alleles). Keep in mind that all the SNPs used in these type of analysis are bi-allelic, so the genotypes can only be homozygous for allele 1, homozygous for allele 2, or heterozygous.

Figure 4

Speed Congenic Timeline (~18 months)

- Start crossing donor (carrier) female with recipient strain male in order to generate F1 carrier males (PI).
- Backcross F1 males to generate ~20 N2 carrier males (~25%) (PI)
- Scan N2 carriers with SNPs and select the best breeders (Service)
- Cross best N2 males with several recipient strain females (PI)
- Generate ~20 N3 carrier males (~25%) (PI)
- Genotype N3 mice for heterozygous SNPs in N2 analysis (Service)
- Cross best N3 males with several recipient strain females (PI)
- Repeat same scheme at N4 (and if necessary at N5)

Table 1A

Mouse Phenome Database (MPD)

Blood – Clinical Chemistry – Enzymes – Alanine Aminotransferase

lanine Aminotransferase (plasma ALT, SGPT) ALT8 [IU/L] at 8 Weeks of Ag

Blood Chemistry Survey of 11 Inbred Strains of Mice (Jaxpheno3 group)

Females	Mean	SD	N	Males	Mean	SD	N
B6.129P2-*Apoe*^{tm1U}	41.7	± 5.48	10 mice	B6.129P2-*Apoe*^{tm1U}	40.4	± 13.0	10 mice
B6D2F1/J	70.2	± 44.0	10 mice	B6D2F1/J	65.8	± 38.5	10 mice
BALB/cByJ	72.8	± 31.7	20 mice	BALB/cByJ	90.9	± 42.5	20 mice
BALB/cJ	68.0	± 26.1	20 mice	BALB/cJ	59.8	± 38.5	22 mice
C3H/HeJ	47.3	± 6.55	10 mice	C3H/HeJ	45.7	± 7.65	10 mice
C57BL/6J	43.1	± 14.6	30 mice	C57BL/6J	57.3	± 39.9	54 mice
CBA/J	38.2	± 10.5	21 mice	CBA/J	41.4	± 8.99	30 mice
DBA/2J	54.7	± 24.6	21 mice	DBA/2J	58.4	± 56.1	20 mice
FVB/NJ	45.0	± 6.90	10 mice	FVB/NJ	76.1	± 23.5	12 mice
NOD.CB17-*Prkdc*^{scic}	40.7	± 7.90	10 mice	NOD.CB17-*Prkdc*^{scic}	64.0	± 21.6	10 mice
NOD/ShiLtJ	36.2	± 9.38	20 mice	NOD/ShiLtJ	62.4	± 47.8	20 mice

From MPD at http://phenome.jax.org/db/q?rtn=docs/home (March 2018)

Table 1B

Mouse Phenome Database (MPD)

Blood – Hematology – Cell Counts – Erythrocytes (RBC)

Red Blood Cell Count (RBC) [n/µL] at 8 Weeks of Age

Hematological Survey of 11 Inbred Strains of Mice (Jaxpheno3 group)

Females	Mean	SD	N	Males	Mean	SD	N
B6.129P2-*Apoe*^{tm1U}	10.4	± 0.272	10 mice	B6.129P2-*Apoe*^{tm1U}	10.4	± 0.281	10 mice
B6D2F1/J	11.1	± 0.280	10 mice	B6D2F1/J	11.3	± 0.464	12 mice
BALB/cByJ	10.5	± 0.253	21 mice	BALB/cByJ	10.8	± 0.377	20 mice
BALB/cJ	10.6	± 0.316	22 mice	BALB/cJ	10.7	± 0.613	21 mice
C3H/HeJ	9.48	± 0.244	13 mice	C3H/HeJ	10.1	± 0.254	11 mice
C57BL/6J	10.8	± 0.477	42 mice	C57BL/6J	10.6	± 0.383	34 mice
CBA/J	9.79	± 0.525	21 mice	CBA/J	10.3	± 0.391	23 mice
DBA/2J	11.6	± 0.526	23 mice	DBA/2J	12.0	± 0.516	20 mice
FVB/NJ	9.80	± 0.310	11 mice	FVB/NJ	10.8	± 0.350	14 mice
NOD.CB17-*Prkdc*^{scic}	9.50	± 0.290	11 mice	NOD.CB17-*Prkdc*^{scic}	9.40	± 0.259	12 mice
NOD/ShiLtJ	9.73	± 0.219	21 mice	NOD/ShiLtJ	10.3	± 0.293	26 mice

From MPD at http://phenome.jax.org/db/q?rtn=docs/home (March 2018)

Table 2: Online resources for laboratory mouse and rat strains

Database	Subject matter	URL
Mouse Genome Informatics	Genome and markers information	http://www.informatics.jax.org/
Ensembl	Genome browser	http://useast.ensembl.org/index.html
Mouse Genomes Project	Genome and markers information	https://www.sanger.ac.uk/science/data/mouse-genomes-project
Mouse Microsatellite Data Base of Japan	Markers information	https://shigen.nig.ac.jp/mouse/mmdbj/top.jsp
Mouse Phenome Database	Phenotype and markers information	http://phenome.jax.org/
International Mouse Phenotyping Consortium	Strain (phenotype) information	https://www.mousephenotype.org/impress/
The global ImMunoGeneTics Web Resource	MHC information	http://www.imgt.org/IMGTrepertoireMHC
Federation of International Mouse Resources	Strain resources	http://www.fimre.org
International Mouse Strain Resource	Strain resources	http://www.findmice.org/
The Jackson Laboratory	Strain resource	https://www.jax.org/
Riken Bioresource Center	Strain resource	http://mus.brc.riken.jp/en/
European Mouse Mutant Archive	Strain resource	https://www.infrafrontier.eu/infrafrontier-research-infrastructure/organisation/european-mouse-mutant-archive
Europhenome mouse phenotyping resource	Strain (genotype) information	http://www.europhenome.org/
MouseMine	Strain information	http://www.mousemine.org/mousemine/begin.do
Mutant Mouse Resource & Research Centers (MMRRC)	Strain information	https://www.mmrrc.org/
Rat Resource & Research Center	Strain resource	http://www.rrrc.us/
The National BioResource Project - Rat	Strain resource	http://www.anim.med.kyoto-u.ac.jp/nbr/Default.aspx
Rat Genome Database	Strain and markers information	http://rgd.mcw.edu/
RatMine	Strain information	http://ratmine.mcw.edu/ratmine/begin.do

Table 3

Microsatellite	BALB/c	C57BL/6	DBA	C3H	AKR
D1Mit24	202	202	218	202	218
D2Mit59	120	146	134	120	146
D3Mit200	127	131	107	131	115
D4Mit32	184	148	142	184	182
D5Mit222	104	104	89	104	89
D6Mit150	140	140	150	150	150
D8Mit155	139	115	151	151	151
D9Mit179	147	147	149	149	151
D11Mit78	80	106	80	80	122
D11Mit228	124	134	114	114	120
D13Mit67	140	152	162	160	162
D13Mit185	152	146	148	152	146
D16Mit139	174	148	174	172	148
D17Mit123	137	133	155	155	155
D18Mit202	143	111	143	143	133

Table 3. Mouse microsatellites. This is just a selection of 15 microsatellites (SSLPs) markers (in 13 chromosomes) that are polymorphic between a group of common classical inbred strains. This list could be used for genetic monitoring of these strains, if we carefully select the markers for each strain combination. The values represent the size of the allele in base pairs (we can observe this after PCR amplification from genomic DNA). The nomenclature for microsatellites is as follows: D [chromosome number] [Lab code] [marker ID], for example, *D18Mit202* is a marker located on chromosome 18, identified at Massachusetts Institute of Technology (MIT) with ID #202.

Standard protocol for PCR amplification of SSLPs using genomic DNA

PCR reaction

Reagents:

Primers (from any commercial provider of oligonucleotides)
Resuspended at 300 µM (stock), then diluted at 30 µM (working solution)
10x PCR buffer with 15mM MgCL2
Taq DNA Polymerase (5U/µl)
Sterile PCR H2O
dNTPs solution, 10mM (combine equal parts dTTP, dGTP, dATP, dCTP)
Inert red dye (5X) for direct gel loading (optional)

Solutions:

It is very practical to prepare a "pre-master mix" with all the reagents but the primers and the Taq polymerase.

Pre Master Mix (100 Reactions)
To use with 1 µl DNA, 4 µl H2O, 0.1 µl Taq and 1µl of each primer in a 25 µl PCR reaction.

1 ml of PCR water
500 µl of 5X red dye
250 µl of 10X PCR buffer with ClMg (15 mM)
50 µl dNTPs (mix of 10 mM each)

To prepare the PCR reactions use this amounts (per DNA sample)

18 µl pre-master mix
1 µl forward primer
1 µl reverse primer
0.1 µl Taq
3 µl H2O
2 µl DNA (at 40 ng/µl)

PCR Cycles

94°C/1 min (1 cycle)
95°C/35 sec; 55°C/45 sec; 68°C/45 sec (40 cycles)
68°C/10 min (1 cycle)

Running and visualizing the bands (electrophoresis)

Reagents:

TBE or TAE running buffer
Agarose NuSieve 3:1 (FMC) or wide range/standard 3:1 (SIGMA)
Ethidium Bromide (stock concentration of 10 mg/mL)
100 bp ladder

Prepare a 4% agarose gel
Add 20µl Ethidium Bromide to 1xTBE running buffer on first use and additional 10µl to refresh for each additional run.
Load 4% gel with 15 µl of each sample.
Include 2 µl of 100 bp ladder.
Run approximately 2 hours at 100 volts.
Visualize gel under UV light, save picture, label and record results.
Size of PCR product varies but is generally between 100-300 bp.

Legislation and 3Rs

1 Legislation

In Europe, animals used for scientific purposes are protected by the Directive 2010/63/EU on the Protection of Animals Used for Scientific Purposes[1] which is supplemented by Commission Recommendation 2007/526/EC of 18 June 2007 on guidelines for the accommodation and care of animals used for experimental and other scientific purposes[2], and the European Convention for the Protection of Vertebrate Animals used for Experimental and other Scientific Purposes, European Treaty Series 123 (ETS 123)[3] issued for signature and ratification by the Council of Europe in 1986. The European legislative framework has been transposed into the national legislations of the Member States of the European Union (MSs). Some MSs may have additional requirements in place at the national or regional level. In 2012 the European Commission published implementing decision 2012/707/EU establishing a common format for the submission of information in accordance with Directive 2010/63/EU[4]. In 2016 a Discussion paper was issued in which the reporting of genetically altered (GA) animals was further explained[5]. In addition, there are recommendations and guidelines from Expert Working Groups (EWGs at the level of the European Commission) and organisations like: FELASA[6], ESLAV[7] and ECLAM[8]. In this context the 'Working document on genetically altered animals' from the EWG for statistical reporting is considered relevant[9]. In the following paragraphs the most relevant aspects of the documents mentioned above for working with GA-animals are summarised[10].

---

[1] The European Parliament and the Council of the European Union. Directive 2010/63/EU of the European Parliament and of the Council of 22 September 2010 on the Protection of Animals Used for Scientific Purposes. *Official Journal of the European Union* 2010; L 276/33-79.

[2] Commission Recommendation 2007/526/EC of 18 June 2007 on guidelines for the accommodation and care of animals used for experimental and other scientific purposes. *Official Journal of the European Union* 2007; L 197/1-89.

[3] European Convention for the Protection of Vertebrate Animals used for Experimental and Other Scientific Purposes. European Treaty Series 123. 1986.

[4] Commission implementing decision of 14 November 2012 establishing a common format for the submission of the information pursuant to Directive 2010/63/EU of the European Parliament and of the Council on the protection of animals used for scientific purposes (notified under document C(2012) 8064) (2012/707/EU).

2 Council of Europe Convention (ETS 123)

ETS 123 is a Convention of the Council of Europe (CoE). It has no legislative power but seeks voluntary cooperation within the member countries of the CoE. Member countries may sign and ratify the Convention. Once ratified the Member country is bound to implement the Convention into its national legislation.

ETS 123 has 38 articles distributed in 12 Parts, and 2 Appendices (A and B). The first Part of the ETS 123 (Articles 1-4) relates to the General Principles, where the scope and definitions are included. Part II (Article 5) focuses on General Care and Accommodation of laboratory animals and refers to the Appendix A that contains the Guidelines for Accommodation and Care of Animals. Appendix A, revised in 2006[11], is one of the most important documents of the European legislative framework because of the impact of its recommendations (for all common species) on housing (cage size especially) and on environmental enrichment and care, which have been partially incorporated as Annex III in the European Directive 2010/63/EU[12]. The minimum cage sizes recommended in the revised Appendix A are significantly larger than those in the original version of 1986, and this is still a controversial issue. The Appendix A includes general recommendations on physical facilities, environment and its control, and care as well as a long species-specific section where recommendations on environment, health, housing, enrichment and care are defined for all the commonly used laboratory animal species. Part III of the ETS 123 (Articles 6-12) relates to the planning and conduct of procedures, stressing (implicitly) the implementation of the principles of the 3Rs [136]. The articles in Part III focus on the use and promotion of alternative methods, choice of species, minimisation of pain and distress, re-use of animals and euthanasia. Part IV deals with the authorization of procedures and persons carrying out procedures (Article 13). Administrative measures for breeding or supplying establishments are defined in Part V (Articles 14-17), which includes registration of establishments and records and identification of animals. Part VI (Articles 18-24) deals with the requirements for user establishments including those related to animal needs, personnel (responsible persons, veterinarian, and training). A list of animals required to be purpose-bred (originated from breeding establishments) is included. The education and training of personnel is specifically

addressed in Part VII (Articles 25-26), and the statistical information in Part VIII (Articles 27-28), which refers to Appendix B containing the statistical tables. Parts VIII-XII (Articles 30-38) relate to legal aspects on the implementation of the Convention.

3 The Directive 2010/63/EU

Directive 2010/63/EU is the result of the revision of Directive 86/609/EEC. The main objectives of 2010/63/EU are: further harmonization of the legislative framework within the EU to create a level playing field; protection of laboratory animals; and explicit implementation of the principles of replacement, reduction and refinement (3Rs principle) [136].

The Directive 2010/63/EU has an introductory part or preamble with 56 recitals that precedes the provisions distributed in 66 Articles (in 6 different Chapters), and 8 Annexes.

The first Chapter (Articles 1-6) relates to the General Provisions, including the scope, definitions, the application of stricter measures, the 3Rs principle, the permitted purpose of experimental procedures and the methods of euthanasia (or "killing"). Article 6 on "methods of killing" refers to Annex IV which lists the accepted methods by species.

Chapter II (Articles 7-11) establishes the provisions on the use of certain groups of animals in procedures (e.g. animals bred for the use in procedures, endangered species, non-human primates, stray and feral animals).

Requirements on planning and the conduct of procedures are specified in chapter III (Articles 12-19). These articles refer to issues such as the choice of methods (by application of the 3Rs principle), application of anaesthesia and analgesia, the mandatory classification of the severity of procedures, the requirements for the re-use of animals, the sharing of organs and tissues and the potential of rehoming or setting free animals. The requirement for the prospective classification of severity of procedures set out by Art. 15 of the Directive is particularly important. Examples of procedure classification are listed in Annex VIII.

Chapter IV (Articles 20-45) is the most extensive. Its first Section (Articles 20-33) focuses on the requirements for breeders, suppliers and users. Articles 22 and 33 refer to Annex III for installations and equipment, care and accommodation respectively. In contrast with the old

Directive, where the provisions of Appendix A were only in the form of recommendations, Directive 2010/63/EU requires the obligatory compliance with these minimum standards.
The second Section of Chapter IV (Art. 34-45) defines the inspections and controls by the competent authorities, and the third Section focuses on the requirements for projects (e.g. mandatory project evaluation and authorisation by the competent authority).
The alternative approaches are addressed in Chapter V (Chapters 46-49). The Chapter VI (Art. 50-66) contains the Final Provisions, such as the requirement for reporting the use of animals in procedures, the safeguard clauses, and the requirements for competent authorities and penalties which had to be observed by the MSs when transposing the Directive into national legislation.

4 Working documents on genetically altered animals and consistent reporting of statistical data
In line with Art. 3 no. 1 of Directive 2010/63/EU not only the creation of a new line of GA animals, but also the maintenance of an existing line is regarded as a procedure as long as it may cause the offspring a level of pain, suffering, distress or lasting harm equivalent to, or higher than that caused by the introduction of a needle in accordance with good veterinary practice. The Working document specifies the consensus reached for the understanding of how procedures involving GA animals are authorised and covered by statistics. It contains an Annex listing key elements of a GA rodent welfare assessment scheme. The Discussion paper has been developed in response to difficulties reported in three areas with the assessment of actual severity, and which were thought likely to result in non-uniform reporting of statistical reporting.

It should be understood that the working document is intended as guidance to assist the MSs, authorities and establishments (users and breeders) to arrive at a common understanding of the provisions stipulated by the Directive. It is, therefore, important that the definitions are clear and well understood:

- ‘Genetically altered animals’ include genetically modified (transgenic, knock-out, and other forms of genetic alteration) as well as naturally occurring or induced mutant animals.

- An animal with ‘a harmful phenotype’ in context of GA-animals is to be understood as an animal that is likely to experience, as a consequence of the genetic alteration pain, distress, suffering or lasting harm equivalent to, or higher than that caused by the introduction of a needle in accordance with good veterinary practice.

5 Project authorisation

In this context a project authorisation is necessary in the following instances:

- creation of a new GA-line until the line is ‘established’. A new GA-line is considered ‘established’ when transmission of the genetic alteration is stable over a minimum of two generations, and an initial welfare assessment is completed.
- use of animals for the maintenance of colonies of GA established lines with a likely harmful phenotype.
- breeding of GA-lines retaining a risk of developing a harmful phenotype (e.g. age of onset of disease or tumours; risk of infection due to a compromised immune system) regardless of the applied refinement (e.g. barrier conditions, culling at an early age); in this case a project authorisation is necessary because the application of refinement techniques does not eliminate the risk.
- use of GA-animals in a procedure requires project authorisation.

GA-lines requiring a specific, intentional intervention to induce gene expression (e.g. chemical induction, mating of Cre with appropriate Lox animals) may be considered as having a non-harmful phenotype until deliberate induction of gene expression. Therefore, their breeding and maintenance does not require project authorisation.

Characterisation, i.e. genotyping using an invasive method such as tail clipping, requires reporting unless the tissue is obtained as a by-product from identification e.g. ear notching. The question whether genotyping should be reported as part of continued use or as first use depends on the purpose of the genotyping. If the genotype of a particular animal is required to be confirmed as a prerequisite for carrying out the further procedure to be carried out, the genotyping of that

animal would be considered the first step in a ‘continued use’. If the genotyping is carried out routinely in a breeding colony of an established line to confirm that the genotype has not varied from the intended genetic background and in order to make it possible to use the animal in another procedure, the latter use is considered as re-use and all such events should be reported separately in the statistics.

6 Statistical reporting

GA animals have to be reported in the following instances:

- when used for the creation of a new line. In addition, those used for superovulation, vasectomy and embryo implantation should equally be reported;
- when used for the maintenance of an established line bred under project authorisation and exhibited pain, suffering, distress or lasting harm due to a harmful genotype.
- when used in other procedures.

On the other hand, genetically unaltered animals i.e. wild type offspring produced as a result of the creation of a new genetically altered line should not be reported.

Similarly, the breeding of animals from a GA-line with a non-harmful phenotype does not require project authorisation, and subsequently no reporting is necessary under the annual statistics.
